# Recurrent and Non-Recurrent Copy Number Variants in Native Americans and a Cosmopolitan Sample in Relation to Alcohol Use Disorder and Other Psychiatric Diseases

**DOI:** 10.1007/s12035-026-05808-w

**Published:** 2026-03-23

**Authors:** Salma M Wakil, Keita Morisaki, Pei-Hong Shen, Dylan G Sucich, Melanie Schwandt, James Fielding Hejtmancik, Cheryl Marietta, Qiaoping Yuan, Nancy Diazgranados, Colin A Hodgkinson, David Goldman

**Affiliations:** 1 Laboratory of Neurogenetics, National Institute On Alcohol Abuse and Alcoholism, Bethesda, USA; 2 Office of the Clinical Director, National Institute On Alcohol Abuse and Alcoholism, Bethesda, USA; 3 Molecular Genetic Ophthalmology Section, Ophthalmic Genetics and Visual Function Branch, National Eye Institute, Bethesda, USA; 4 Department of Biology, Johns Hopkins University, Baltimore, USA

**Keywords:** Copy number variation, Alcohol use disorder, Substance use disorder, Psychiatric disease, Velocardiofacial syndrome

## Abstract

Copy number variants (CNVs) can alter disease susceptibility by gene deletion, duplication, and other mechanisms, and have been implicated in neuropsychiatric diseases. However, their rarity or de novo nature impedes linkage analysis. Therefore, we identified recurrent CNVs (rCNVs) in Native Americans with low genetic admixture and high prevalence of alcohol use disorder (AUD) and other psychiatric disorders. Large (> 200 kb) rCNVs were abundant in Plains Indians (PI) and Southwest American Indians (SWI), almost all carrying at least one rCNV, with some CNVs found in both geographically and linguistically distinct tribes. In patients carrying rCNVs, gene deletions led to haploinsufficiency, and duplications led to increased gene dosage. Haplotype analysis revealed a common chromosome 6p21.33 recurrent CNV (rCNV) that persisted in Native Americans for at least 750 generations, leading to haploinsufficiency of at least two genes. Gene-based CNV burden and CNV count did not predict AUD or other psychiatric disorders. However, an rCNV, found in PI and duplicating three genes within the 22q11.2 velocardiofacial syndrome region, showed nominally elevated odds ratios in generalized linear mixed models accounting for kinship as a random effect and age and sex as fixed covariates. For AUD, the odds ratio was 3.19 (95% CI 1.12–9.07, *p* = 0.03), and for psychiatric diagnosis, the odds ratio was 4.80 (95% CI 1.37–16.82, *p* = 0.014). These associations, calculated with adjustment for relatedness, did not remain statistically significant after correction for multiple testing, illustrating the challenge of linking CNVs to behavior even if they are recurrent and potentially of large effect. The effects of most CNVs are undetectable via genome-wide association studies with single SNPs, and in cosmopolitan populations, most CNVs are nonrecurrent, sometimes affecting similar genomic regions but differing in their properties, and origins. Recurrent CNVs having ancient origins are prevalent in Native American populations providing an opportunity to examine their relationships to disease risk. However, we observed that neither gene-based CNV burden nor individual rCNVs did not predict AUD or other psychiatric outcomes, after adjustment for the numbers of rCNVs tested.

**Supplementary Information:**

The online version contains supplementary material available at 10.1007/s12035-026-05808-w.

## Background

Copy number variations (CNVs) are one of the most phenotypically significant classes of genomic variation. These structural variants range in size from indels of single nucleotides to large chromosomal regions. CNVs are far less common than single nucleotide polymorphisms (SNPs) that number approximately 22 million [1] or the rarer single nucleotide variants (SNVs) that are even more numerous. However, because of their larger size, CNVs contribute a comparable amount of nucleotide heterozygosity. On average, individuals may carry a burden of 1–2 deleted or duplicated genes attributable to large CNVs [2]. CNVs intersecting gene regions can modify disease susceptibility through multiple mechanisms, including gene dosage alterations resulting from deletions or duplications, disruption or fusion of genes at breakpoint regions, positional effects on gene regulation, unmasking of recessive alleles, and structural changes that alter transcriptional or post-transcriptional regulation of gene expression [3–5]. CNVs involving large chromosomal segments that duplicate or delete genes are thought to be subject to strong negative selection and to therefore be rare and persistent for only a few generations. Many are only observed as de novo, sporadic mutations. For example, velocardiofacial syndrome (VCF), caused by large, multigene, deletions at 22q11.2, occurs de novo in 90% of cases [6, 7]. Structural variation due to different mutational origins contributes to variable expressivity and penetrance.

CNVs are often described as recurrent or non-recurrent based on their breakpoint structure and mutational mechanisms. Recurrent CNVs (rCNVs) arise through non-allelic homologous recombination (NAHR) between flanking low-copy repeats, producing highly similar breakpoints in unrelated individuals and can represent homoplasic events. Non-recurrent CNVs, in contrast, have variable breakpoints and always arise via distinct mutational events. Large CNVs have been robustly associated with diverse disorders including autism, schizophrenia, type I diabetes, congenital abnormalities, and neurodegenerative diseases [8–12]. These CNV-phenotype associations have been discovered for CNVs that are individually rare and of different mutational origins, but that tend to recur in regions that represent recombination hotspots, sites of NAHR, or locations of L1 retro transposition [13, 14].

The psychiatric disease burden attributable to CNVs is unknown but may be substantial. Including 22q11.2 deletions, CNVs contribute as much as 10% of the genetic risk of schizophrenia [2, 15–17]. The CNVs contributing to schizophrenia are primarily non-recurrent, occurring at the same chromosomal regions but exhibiting variable breakpoints and representing separate mutational events. Similarly, numerous CNVs have been implicated in developmental delay, intellectual disability (ID), and autism, contributing an estimated 14% of the genetic risk to these interrelated disorders [18–20].

The role of CNVs has been investigated in other neuropsychiatric disorders but is still largely unknown. Recently, the ability of CNVs to alter brain function and an approach to systematically screening for functional consequences of CNVs was demonstrated using single-cell spatial transcriptomics [21]. However, psychiatric disorders are multifactorial, and their vulnerability can be influenced by many loci of small molecular and downstream phenotypic effects, as shown by genome‐wide association studies (GWAS). A meta-analysis found significant enrichment of smaller CNVs located primarily in intergenic regions and enhancers in individuals with major depressive disorder (MDD), suggesting a role for CNVs in altering gene expression and increasing MDD risk [22]. In the large UK Biobank sample, known neurodevelopmental CNVs conferred a 1.25–1.3 OR (odds ratio) for depression, with no effect detected for other CNVs, perhaps because of either the rarity of these CNVs or their effect sizes. CNVs at 1q21.1, 16p11.2, and the 15q11-13 Prader-Willi region were associated with self-reported depression. Thus, the CNVs implicated in depression seem to be pleiotropic, conferring risk to other phenotypes in addition to depression [23–27]. In a smaller sample of children, large CNVs were associated with anxiety and depression [28]. Children with attention deficit hyperactivity disorder (ADHD) were more likely to carry de novo CNVs [29].

The relationship of CNVs to alcohol use disorder (AUD) or other substance use disorder (SUD) is unknown. Remarkably, in a large, epidemiologically representative sample (NESARC III), DSM-5 AUD affected 29.1% of the US population on a lifetime basis [30] and the 1-year prevalence of AUD was estimated to be 13.9% (www.niaaa.nih.gov). Unlike other psychiatric disorders that may be triggered by exposures, AUD and other SUDs are dependent on a chosen exposure. Nevertheless, these disorders are moderately to highly heritable, and cross-transmitted, apparently because of shared genetic influences on processes such as reward, executive cognition, and negative emotion, that are common to the vulnerability and progression of different addictive disorders, as well as other psychiatric disorders [31]. As is true for other psychiatric disorders, many genes of small effect contribute to AUD and other SUDs, as demonstrated by GWAS. Furthermore, the advent of polygenic scores has confirmed that SUDs are cross transmitted with each other and other phenotypes as well [32, 33].

Regarding the contribution of specific CNVs to AUD, an association study linked CNVs at the16q12.2 and 9p21.2 regions to AUD. However, these effects, although large in magnitude, were not replicated in other studies that instead implicated CNVs in other regions [34]. Notably, the number of people carrying a CNV at any region was always small, and furthermore, those CNVs were molecularly distinct in different individuals. The observed role of CNVs in schizophrenia and autism suggests that it would be valuable to study large numbers of cases and controls carrying CNVs affecting the same region, and if possible, carrying the identical rCNV. We performed these studies in two Native American Indian tribes with high rates of AUD and other psychiatric disorders, comparing the effects of rCNVs and overall CNV burden to a cosmopolitan sample of AUD cases and controls collected at the NIH Clinical Center (NIH CC) which served as a comparator cohort.

## Results

**Fig. 1:**
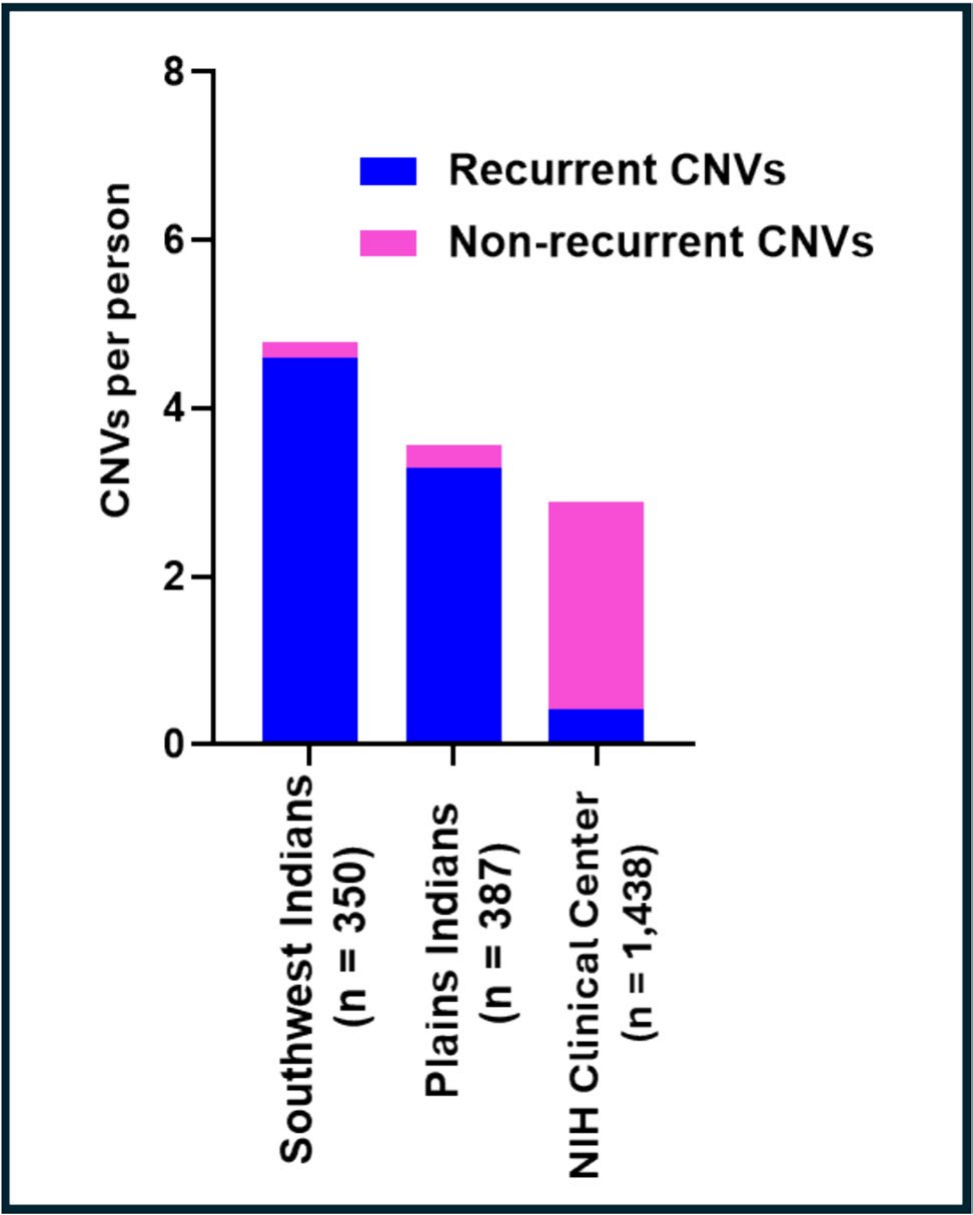
The profile of nonrecurrent and recurrent CNVs (rCNVs) in three psychiatrically characterized samples: Plains Indians (*n* = 387), Southwest Indians (*n* = 350), and NIH Clinical Center (*n* = 1438). The average number of recurrent (blue) and non-recurrent (pink) CNVs per individual is shown. CNVs were identified from SNP array data and classified as recurrent if observed in multiple individuals while nonrecurrent CNVs were unique to single individuals

**Fig. 2:**
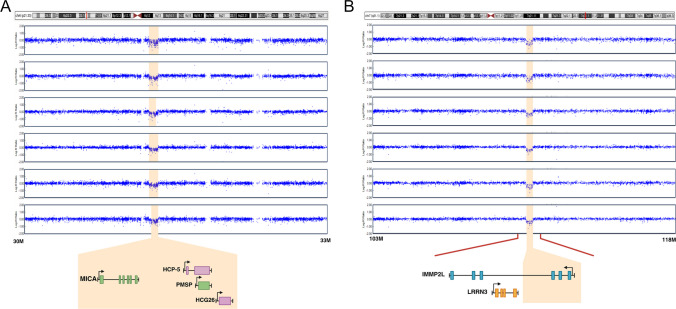
Signal intensities of two rCNVs causing deletions. The 6p21.33 (**A**) and 7q31.1 (**B**) rCNVs identified in 154 and 22 individuals from two Native American populations led to decreased log R ratio (LRR) of SNPs within the respective regions. Genes within the 98 kb deletion of 6p21.33 were *MICA*, *HCP5*, *PMSP*, and *HCG26*, and within the 156 kb region of 7q31.1, *IMMP2L* was partly deleted

**Fig. 3:**
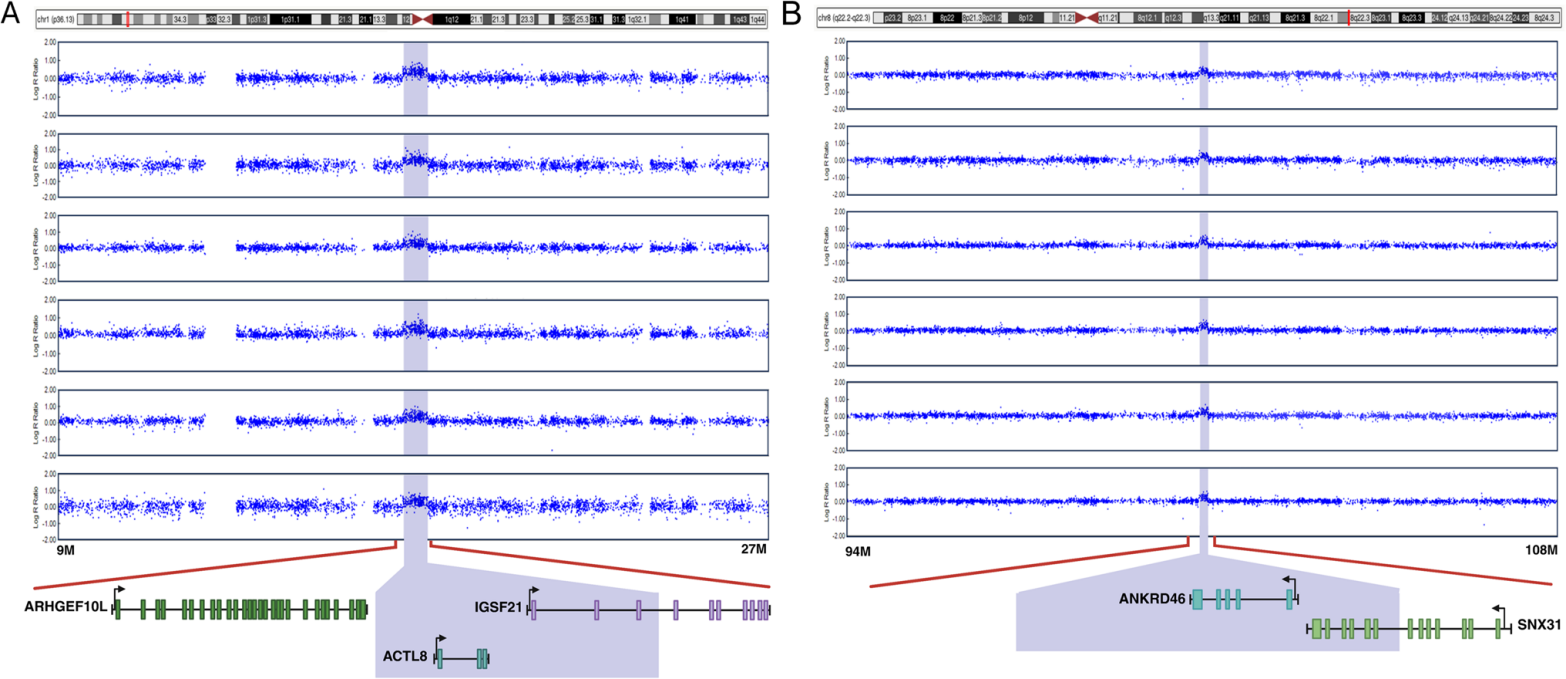
Signal intensities of two rCNVs causing duplications. The 1p36.13 (**A**) and 8q22.2-q22.3 (**B**) rCNVs identified in eight and six individuals from two Native American populations led to increased log R ratio (LRR) of SNPs within the respective regions. Within the 623 kb deletion of 1p36.13, *ACTL8* was duplicated and *IFSF21* was partly duplicated. Within the 190 kb duplication, *ANKRD46* was duplicated and *SNX31* was partly duplicated (Figs. 2 and 3 were created in https://BioRender.com)

Coincidentally, the PI, SWI, and NIH CC samples had similar ratios of AUD cases to controls, the fractions of AUD cases being 0.60, 0.71, and 0.62, respectively (Table S1). These high prevalences reflect the abundance of AUD in the PI and SWI communities, and the ascertainment bias of the NIH CC sample for research on AUD. Additionally, these cohorts had high prevalences of other psychiatric disorders (Tables 1 & S1). The overlap between CNVs detected by both CNV Partition and Penn CNV, which were used in subsequent analyses, was high (0.956). There were a total of 1384 CNV events in PI, 1696 in SWI, and 4179 in the NIH CC sample, translating to an average of 3.6 CNVs per person in PI, 4.8 in SWI, and 2.9 in the NIH CC sample (Fig. 1). Of these CNV events, 939 (68%), 1054 (62%), and 1143 (27%) were deletions, and reciprocally 445 (32%), 642 (38%), and 3036 (73%) were duplications in PI, SWI, and the NIH CC sample, respectively (Figs. 2–3). Thus, deletions were more common in the Native Americans (*p* ≪ 0.001). Accordingly, differences in CNV class distributions between the Native American samples and the NIH Clinical Center sample should be interpreted cautiously and are intended for contextual comparison rather than formal cross-cohort inference. When CNVs were stratified by class, deletions comprised 70% and 63% of rCNVs in PI and SWI, respectively, compared with 20% in the NIH CC sample. Rare CNVs showed near-equal deletion and duplication rates in PI (50 vs 50%) and SWI (47 vs 52%), whereas rare CNVs in the NIH CC sample were predominantly duplications (71.5%). The average size of all CNVs (including both deletions and duplications) was 317 kb in PI, 192 kb in SWI, and 269 kb in the NIH CC sample.

**Table 1:** Demographic characteristics of Plains Indians, Southwest Indians and NIH CC samples

Populations	Plains Indians	Southwest Indians	NIH CC
N	387	350	1438
Age (SD)	42.0 (14)	35.9 (13.1)	40.6 (13.3)
Sex (%) Male Female	169 (44) 218 (56)	150 (43) 200 (57)	851 (59) 587 (41)

**Fig. 4:**
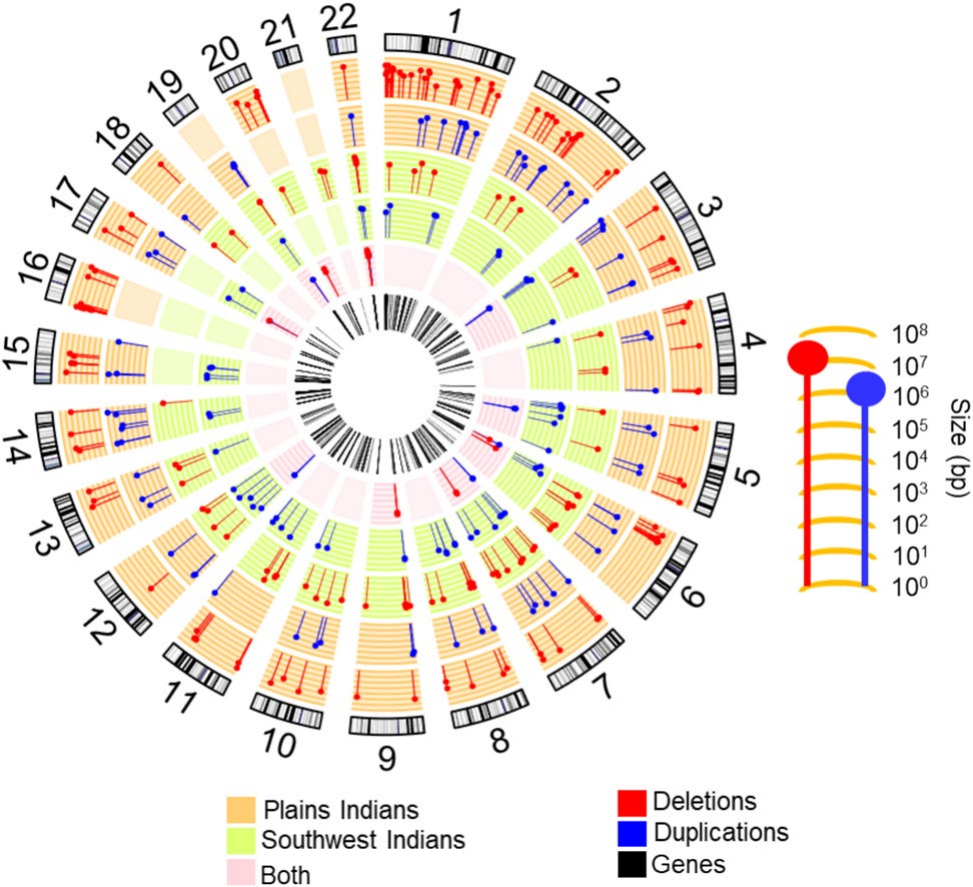
rCNVs are distributed across the genome in two Native American tribes. Circos tracks are as follows (from outer to inner): (1) locations of rCNVs on the GRch37 karyotype: deletions (red) and duplications (blue); (2, 3) orange tracks- rCNVs observed in Plains Indians, the length of symbols (**j**) corresponding to sizes, ranging up to 10 Mb (10^7^ bp), as shown; (4, 5) green tracks- rCNVs observed in Southwest Indians, the lengths of symbols (**j**) again corresponding to sizes; (6) pink track- rCNV deletions (red) and duplications (blue) common to both Native American populations; and (7) the bars in the innermost track correspond to genes deleted, duplicated, or partially deleted or duplicated by rCNVs

Compared to the NIH CC sample, Native Americans had proportionately fewer non-recurrent CNVs but harbored far more rCNVs (Fig. 4). In total, 295 distinct rCNVs were identified, with 165 rCNVs in SWI, 114 in PI rCNVs, and 16 “shared” by both (Tables 2, S3–S4). To contextualize the recurrence of the most frequent rCNVs (*n* = 16; Table S2), we assessed their allele frequencies in global reference populations using the Genome Aggregation Database-Structural Variation (gnomAD-SV). Globally, most rCNVs were rare, with observed allele frequencies in different populations ranging from 0.00005 to 0.013, and several variants were not represented in gnomAD-SV, likely reflecting ancestry underrepresentation, technical differences in CNV detection, or differences in breakpoint definitions. In contrast, in the Native American cohorts, allele frequencies of rCNVs ranged up to 0.14 in SWI and to 0.10 in PI.

**Table 2:** Shared recurrent and Non-recurrent CNVs detected in plains Indians and Southwest Indians

**Cytogenetic region**	**CNV type**	**Start (bp)**	**End (bp)**	**Size (Mb)**	**Carrier frequencies**		**Genes**
					**PI**	**SWI**	
13q31.1	Deletion	84102440	84157927	0.05	80 (0.20)	-	*Non-genic region*
		84101480	84157927	0.05	-	51 (0.14)	
6p21.33	Deletion	31355318	31451476	0.09	60 (0.15)	-	*MICA, HCG-26 HCP-5, PMSP*
		31355318	31453640	0.09	-	93 (0.26)	
3p25.2	Duplication	12610706	12792622	0.18	43 (0.11)	-	*RAF1, TMEM40*
		12628920	12806123	0.09	-	19 (0.05)	
22q11.23-q12.1	Duplication	25661725	25910667	0.24	25 (0.06)	-	*LRP5L, CRYBB2P1*
		25650406	25910667	0.26	-	79 (0.22)	
20p12.1	Deletion	14758111	14830453	0.07	18 (0.04)	-	*MACROD2*
		14815778	15171838	0.35	-	2 (0.005)	
9p24.3	Duplication	526772	704075	0.17	7 (0.01)	-	*KANK1*
		483018	489338	0.006	-	2 (0.005)	
17p11.2-p11.1	Duplication	21704418	22242355	0.53	19 (0.04)	-	*MTRNR2L1*
		21539613	22242355	0.70	-	18 (0.05)	
12p13.31	Duplication	7996890	8123306	0.12	4 (0.01)	-	*SLC2A14, NANOGP1, SLC2A3*
		8000912	8114429	0.11	-	2 (0.005)	
7q31.1	Deletion	111085618	111242161	0.15	6 (0.01)	-	*IMMP2L*
		110971919	111309224	0.33	-	7 (0.02)	
19q13.42	Deletion	53932295	54014178	0.08	2 (0.005)	-	*ZNF761, ZNF813*
		53932295	54011384	0.07	-	3 (0.008)	
13q33.2	Deletion	105918084	106007135	0.08	2 (0.005)	-	*Non-genic region*
		106219874	106307349	0.08	-	20 (0.05)	
4q12	Duplication	58604281	58758826	0.15	1 (0.002)	-	*Non-genic region*
		58604281	58770663	0.16	-	7 (0.02)	
7q11.23	Duplication	76066189	76557212	0.49	1 (0.002)	-	*MULTIGENIC*
		76131646	76639871	0.50	-	6 (0.01)	
2q21.1	Deletion	132077379	132311088	0.23	1 (0.002)	-	*MULTIGENIC*
		132057166	132298468	0.24	-	5 (0.01)	
12q14.2	Duplication	63947694	64129108	0.18	2 (0.005)	-	*DPY19L2*
		63946056	64118558	0.17	-	2 (0.005)	
1p13.3	Deletion	109705022	109832283	0.12	2 (0.005)	-	*CELSR2*
		109789795	109820919	0.03	-	5 (0.01)	

We tested whether the admixture of individual Native Americans predicted their rCNV burden. Overall, admixture of both Native American populations with non-Native Americans was low. However, we observed no correlation between level of admixture and number of rCNVs that individuals carried in either population (Figure S3). The absence of relationship of abundance of rCNVs to admixture was also borne out at the level of individual rCNVs, wherein some rCNVs were more frequent in SWI, others more frequent in PI, and some rCNVs were observed in only one population. Although a few rare rCNVs were present in the NIH CC sample, none overlapped with those observed in Native American populations and rCNV burden was comparatively small in the NIH CC sample (Fig. 1).

**Fig. 5:**
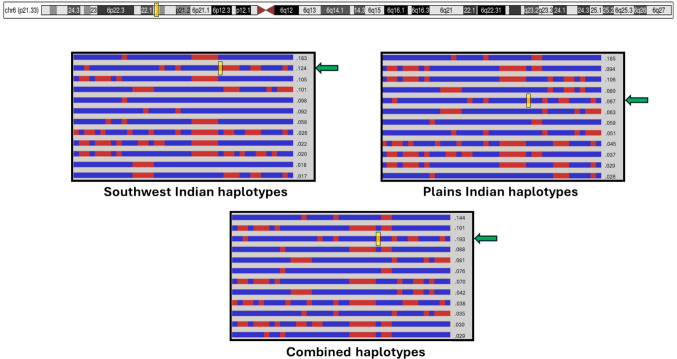
Distinct haplotypes shared by 6p21.33 rCNV chromosomes in both Southwest Indians and Plains Indians. This figure shows distinct shared haplotypes in the region of the 6p21.33 rCNV. The yellow boxes denote the location of the 6p21.33 deletion, and the green arrows show the haplotypes containing the 6p21.33 rCNV. Haplotype analyses were conducted for multiple recurrent CNVs; the 6p21.33 rCNV is shown here as a representative example due to its high carrier frequency in both Native American cohorts. The frequency of the CNV haplotype (and CNV) was 0.124 in Southwest Indians (*n* = 350) and 0.067 in Plains Indians (*n* = 387)

In the Native American cohorts, we observed that each rCNV occurred on a characteristic haplotype, as confirmed by haplotype analyses performed for each rCNV, thus confirming their common origin, rather than being CNVs that happened to recur in the same chromosomal region and to share the same boundaries (Fig. 5 and Figure S8 show representative examples). This consistency of haplotype background included rCNVs shared to both Native American populations, although the genotyping arrays were different, and therefore the exact SNPs constituting the conserved haplotypes differed between PI and SWI. Because of array resolution, exact CNV breakpoints remain approximate (within 10 kb), and thereby haplotype consistency is important to support their identity as recurrent.

**Fig. 6:**
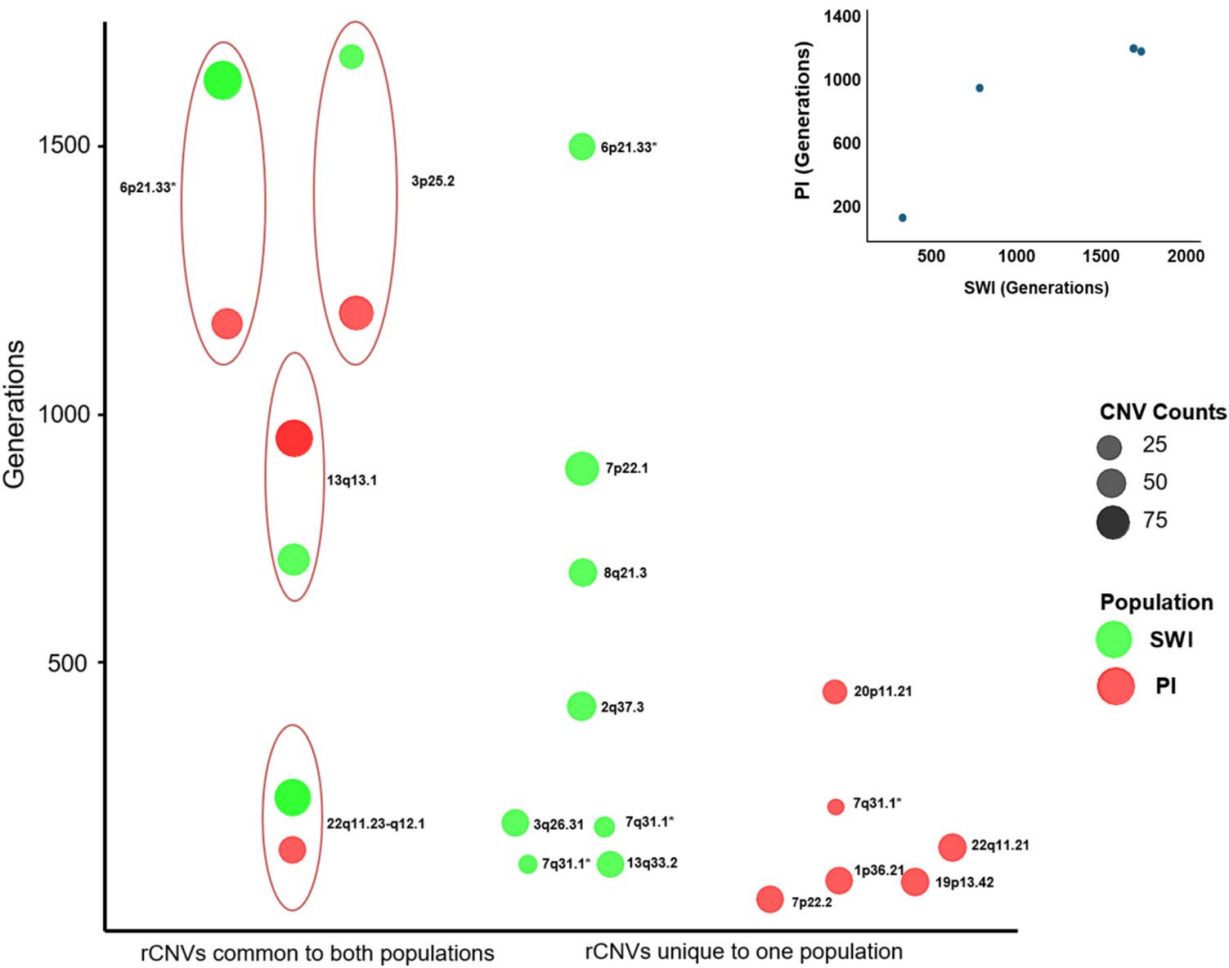
Coalescence times, in generations, of rCNVs present in one or both Native American populations. Shown are four rCNVs present in both SWI and PI (left), eight rCNVs detected only in SWI (center), and six rCNVs detected only in PI (right). Coalescence times were computed using the Gamma method based on the first recombination event observed 5’ and 3’ to the rCNV and using local average recombination rates. As shown in the inset, coalescence times for the four rCNVs identified in both Native American populations tended to correlate although the *p*-value was not computed because *n* was < 5 (* denotes different rCNVs at 7q31.1)

As just mentioned, the consistency of haplotype backgrounds, along with common breakpoints, strongly suggested that each rCNV derived from a common ancestor, whether by mutation occurring in Native Americans or a CNV introduced by an ancestral founder. Notably, 16 rCNVs were shared between these two geographically separated and linguistically distinct Native American populations. The haplotype coalescence times of the rCNVs were calculated via the Gamma method based on sizes of the conserved haplotypes on which the rCNVs resided and by using the recombination rates local to the chromosomal regions (Fig. 6). All coalescence time estimates are reported with confidence intervals to reflect uncertainty arising from haplotype length estimates and variation in recombination rates. The rCNVs observed in both Native American populations were inferred to be ancient, with point estimates suggesting presence in ancestors of the PI and SWI for hundreds of generations to more than a thousand generations, as required for recombinants to be likely to arise within the 5’ and 3’ flanking haplotypes close to the CNVs themselves. The difference in ages of rCNVs observed in both Native American populations versus those observed in only one was statistically significant (Mann–Whitney *U* test, *p* = 0.01). Several of the rCNVs shared by these two Native American populations had upper confidence bounds extending beyond 10,000 years ago (Fig. 6), representing either de novo mutations in early Native Americans or origins in a common Asian ancestor. The rCNVs that were more abundant tended to be more ancient (*r* = 0.445, slope = 0.0194, *p* < 0.05).

In genomic location, the rCNVs were broadly distributed across the autosomes (Fig. 4). Overall, 75% of rCNVs identified in Native Americans contained RefSeq genes, suggesting that many of these rCNVs may directly alter gene expression. Additionally, many of the non-gene containing rCNVs were still proximal to genes. The genes deleted, duplicated, or potentially impacted by the rCNVs are listed in Table 2, with more detailed information provided in Tables S2–S4. Consistent with their ancient origins, rCNVs were observed across multiple branches of the extended pedigrees within each cohort rather than being restricted to any single lineage. Carriers of any particular rCNV did not exhibit systematically higher kinship coefficients compared to noncarriers. Although related individuals carrying the same rCNV were observed, the overall distribution of pairwise kinship coefficients was sharply peaked near zero and largely overlapped between carriers and noncarriers, consistent with the underlying pedigree structure of the cohorts (Figure S1; see also Figure S2 selected for high frequency rCNV).

### Functionality of the 6p21.33 rCNV, and Other rCNVs

We evaluated the functionality of the 96 kb deletion at 6p21.33 (allele frequency of 0.077 in PI and 0.121 in SWI) by transcriptome analysis of five PI lymphoblastoid cell lines (LCLs) heterozygous for this rCNV versus four noncarriers. This analysis also enabled us to test, on a more limited basis, the functionality of other rCNVs and CNVs that were heterozygous in the nine LCLs, and in instances where genes duplicated or deleted by the CNVs were ordinarily expressed in LCLs. We evaluated both *cis* and *trans* consequences of the 6p21.33 rCNV for mRNA expression. Two of the genes within the region deleted by the 6p21.33 rCNV, *MICA* and *HCG-26*, were expressed at measurable levels, and their expression was reduced by approximately 50% in individuals heterozygous for the deletion (Figure S4), consistent with a gene dosage effect. Consistent with other reports, expression of the two other genes within the deleted region, *HCP-5* and *PMSP*, was not detected. The uncompensated reduction of *MICA* and/or *HCG-26* expression appears to have led to *trans* effects on the expression of other genes. Differential gene expression analysis identified 473 genes (|FC|> 1.25; nominal *p*-value < 0.05), of which 202 were upregulated, and 271 were downregulated, and among these, 34 remained significant after FDR correction (Figure S5). Given the small sample size and variability inherent to LCLs, these *trans* effects should be interpreted cautiously and considered preliminary. The expression levels of ten additional genes were potentially impacted in direct (*cis*) fashion by heterozygous duplications or deletions in these nine LCL lines. Each of these additional rCNVs was represented in only one or two heterozygous cell lines. Therefore, to test for *cis* effects on expression, we normalized levels of expression of the genes these rCNVs contained to the non-CNV homozygote and thereby performed a combined analysis of the *cis* effects of the rCNVs on gene expression. As shown in Figure S6, heterozygous deletions reduced expression of genes by approximately 50%, whereas duplications increased expression by approximately 50% although sample sizes were too small for statistical inference.

### Associations of Individual rCNVs

In this study, there was insufficient power to detect small-risk effects of individual loci regardless of whether the loci were rCNVs or other types of genetic variation. We limited our analysis to rCNVs with frequencies > 2.2%, consistent with our power calculations. This allowed detection of large effect sizes (OR ≈10) while providing some ability to observe rCNVs with smaller ORs, albeit with reduced power. To detect large effects of rCNVs on AUD and “Any psychiatric Disorder,” we tested individual rCNVs observed in ≥ 25 carriers in PI (10 rCNVs), or ≥ 22 carriers in SWI (12 rCNVs), with three rCNVs shared across both populations. An rCNV duplication at 22q11.21, observed only in PI, was associated with higher odds of AUD and “Any psychiatric disorders.” In generalized linear mixed models accounting for relatedness, age, and sex, carriers of this CNV had nominally higher odds of AUD (OR = 3.19 [95% CI 1.12–9.07], *p* = 0.03) and “Any psychiatric disorders” (OR = 4.80 [95% CI 1.37–16.82], *p* = 0.01). None of the rCNV associations remained statistically significant after Bonferroni correction. Other rCNVs also followed similar trends, and intriguingly with similar effects of the rCNVs to increase odds of psychiatric disease, but without reaching statistical significance. Deletions at 19q13.42 were enriched in individuals with AUD (OR = 1.31 [0.51–3.32], *p* = 0.56) and with “Any psychiatric disorder” (OR = 1.66 [0.61–4.48], *p* = 0.31). Similarly, deletions at 7p22.2 showed higher odds for AUD (OR = 1.85 [0.72–4.78],* p* = 0.19) and for “Any psychiatric disorder” (OR = 1.46 [0.57–3.71], *p* = 0.42). In addition, deletions at 8p23.2 were more frequent among cases with AUD (OR = 1.91 [0.73–5.01], *p* = 0.18) and for “Any psychiatric disorder” (OR = 1.88 [0.71–5.06], *p* = 0.21). Among SWI, some of the 12 tested rCNVs, such as deletions at 2q37.3 (OR = 2.50 [0.91–6.85], *p* = 0.07) and 7p36.1 (OR = 1.76 [0.57–5.43] *p* = 0.32), exhibited elevated ORs for AUD with wide confidence intervals, suggesting potential associations that may require larger sample sizes for validation. Notably, none of the three rCNVs observed in both populations reached statistical significance for either AUD or “Any psychiatric disorder” (Table 3). As a secondary exploratory analysis outside our pre-specified frequency threshold for formal testing (≥ 20 carriers, corresponding to ≥ 5 expected counts per cell), we examined deletions at 20p12.1, which were more frequent in PI (*n* = 16 carriers) than in SWI (*n* = 2). In PI, this deletion was associated with a comparatively large effect estimate for AUD (OR = 4.68 [1.00–21.9], *p* = 0.04) and a suggestive association with “Any psychiatric disorder” (OR = 3.70 [0.80–17.1], *p* = 0.09). These ORs are numerically larger than those typically reported in GWAS of psychiatric disorders, although the confidence intervals are wide and the estimates are imprecise. While these associations did not remain statistically significant after Bonferroni correction, they may merit further study because of the elevated ORs.

**Table 3:** rCNV associations with AUD and “Any Psychiatric Disorders” in Plains Indians (PI) and Southwest Indians (SWI)

**CNV region**	**Dataset presence**	**AUD**			**Any psychiatric disorder**			**No. of carriers**
		**OR**	**CI (95%)**	*p*-value*	**OR**	**CI (95%)**	*p*-value*	
6p21.33_del	PI & SWI	1.09	0.71–1.69	0.67	1.15	0.71–1.84	0.56	153
13q31.1_del	PI & SWI	1.27	0.79–2.02	0.31	1.28	0.77–2.12	0.32	127
22q11.23_dup	PI & SWI	0.74	0.44–1.23	0.25	0.85	0.49–1.50	0.59	97
11q11_del	PI	1.12	0.65–1.94	0.66	1.05	0.60–1.81	0.86	83
1q21.3_del	PI	1.04	0.57–1.91	0.88	1.05	0.57–1.95	0.85	65
3p25.2_dup	PI	1.08	0.47–2.52	0.84	1.01	0.43–2.35	0.97	29
9p23_dup	PI	0.80	0.33–1.94	0.63	0.78	0.32–1.89	0.59	27
19q13.42_del	PI	1.31	0.51–3.32	0.56	1.66	0.61–4.48	0.31	27
22q11.21_dup	PI	3.19	1.12–9.07	0.03	4.80	1.37–16.8	0.01	27
7p22.2_del	PI	1.85	0.72–4.78	0.19	1.46	0.57–3.71	0.42	26
22q11.22_del	PI	0.52	0.21–1.25	0.14	0.89	0.36–2.18	0.81	26
8p23.2_del	PI	1.91	0.73–5.01	0.18	1.88	0.71–5.06	0.21	25
6q14.1_del	PI	1.36	0.53–3.51	0.51	1.43	0.54–3.79	0.46	25
7p22.1_del	SWI	1.86	0.76–4.27	0.17	1.34	0.51–3.52	0.54	47
7p22.3_dup	SWI	0.92	0.37–2.29	0.86	1.02	0.36–2.89	0.96	36
2q37.3_del	SWI	2.50	0.91–6.85	0.07	2.20	0.62–7.82	0.22	34
16p13.3_del	SWI	1.02	0.39–2.61	0.96	0.81	0.29–2.22	0.69	32
8q21.3_del	SWI	0.67	0.26–1.68	0.39	0.44	0.17–1.12	0.08	31
3q26.31_dup	SWI	0.71	0.28–1.81	0.48	0.57	0.21–1.50	0.25	30
4p16.3_del	SWI	1.02	0.34–3.04	0.96	0.35	0.12–0.98	0.04	23
6p21.33_del	SWI	1.24	0.41–3.79	0.69	0.93	0.28–3.12	0.91	23
7p36.1_del	SWI	1.76	0.57–5.43	0.32	0.93	0.28–3.01	0.90	23
13q12.11_dup	SWI	0.64	0.22–1.80	0.39	0.89	0.27–2.96	0.86	22
17q12_dup	SWI	0.59	0.20–1.68	0.32	1.19	0.32–4.45	0.78	22
19q13.41_dup	SWI	0.75	0.26–2.14	0.60	0.81	0.24–2.68	0.73	22

^*^Nominal *p*-values from adjusted mixed-model analysis; none remained significant after multiple-testing correction

### CNV Burden and Psychiatric Disease

To evaluate consequences of CNV burden, we combined the PI and SWI data and also examined the effect of CNV burden in the NIH CC sample (Table S1). Given differences in phenotyping and genotyping platforms, the NIH CC cohort was analyzed in parallel and not pooled with the Native American samples for primary inference. Comparing the rank order of cases and controls, there was no significant effect of CNV burden on either AUD or “any psychiatric diagnosis.” CNV burden was assessed using aggregate measures including CNV count and gene-based CNV burden (gene load) without stratification by CNV class, size, or gene-level constraint without covariate adjustment; results should therefore be interpreted as exploratory. The gene-based CNV burden was numerically higher in AUD cases when PI and SWI datasets were combined (mean ± SD 15.27 ± 59.2) compared to non-AUD controls (13.36 ± 50.17), but the difference was not statistically significant. In secondary analyses, for post-traumatic stress disorder (PTSD), CNV burden was higher whether measured by number of CNVs (6.51 ± 6.93 vs 4.02 ± 3.28) or gene-based CNV burden (31.41 ± 86.37 vs 13.16 ± 52.64). However, this difference did not survive correction for multiple testing, and confidence intervals were wide.

## Discussion

CNVs deleting or duplicating genes are not rare. In this study, among 387 PI, 350 SWI, and 1438 individuals from the NIH CC, we identified only 145 individuals out of 2175 (12, 10, and 123 respectively), in whom a CNV did not alter the copy number of at least one gene. Differences in ascertainment strategy and diagnostic instruments between the NIH CC cohort and the Native American samples limit direct cross-cohort comparisons, and interpretations emphasizing population-specific patterns are therefore focused primarily on the PI and SWI cohorts. Because CNV breakpoints are inferred from array data, exact boundaries are approximate, with haplotype sharing used to support the biological identity of rCNVs. Large CNVs (> 200 kb) deleting and duplicating genes are likely to be consequential and as recently demonstrated have been explored for several CNVs via transcriptome analysis of postmortem brain [21]. Here, we showed that the haploinsufficiency caused by deletion or gene excess due to duplication was not compensated for a 6p21.33 rCNV in the MHC region, nor in several other rCNVs harboring genes expressed in LCLs. Indeed, dosage compensation, the mechanism by which organisms balance gene expression after deletions or duplications, appears to be the exception, rather than the rule, in eukaryotes, as demonstrated by genes deleted in *Saccharomyces* [35] or duplicated in humans via Trisomy 21. Thus, the lack of compensation observed for these rCNVs suggests that gene dosage changes are likely to have functional consequences. For the 6p21.33 rCNV, the only rCNV we tested using multiple cell lines stratified to be heterozygotes or noncarriers for the rCNV, we also observed a cascade of *trans* effects.

The importance of the lack of dosage compensation is amplified by the fact that many of the genes deleted or duplicated in the rCNVs have been implicated in heritable diseases (Table S2). Potentially, large CNVs could contribute to the so-called missing heritability of psychiatric diseases and other phenotypes, and furthermore, de *novo* CNVs represent a genetic origin of variance that, in many or even most instances, would not contribute to heritability. Although de novo CNVs have been associated with phenotypic consequences in other populations, our study focused on rCNVs in Native Americans and their functional implications. A well-characterized example is the common deletion of the *GSTM1* (glutathione s-transferase Mu 1) which reduces enzymatic activity and heightens oxidative stress, especially in combination with xenobiotics such as are found in tobacco smoke. The *GSTM1*-null genotype promotes cancers, metabolic, and autoimmune disorders, but absence of GSTM1 activity can itself be partially compensated by the overexpression of other GST family members [36]. Often, linkage of CNVs to phenotypes is impeded by their low frequencies, and by divergences in molecular effects arising from differences in CNV breakpoints.

The rCNVs we identified in two Native American Indian tribes may be recurrent due to founder effects, population bottlenecks, and/or relatively small effective breeding sizes, these representing interrelated but somewhat distinct possibilities. Indeed, both of these Native American populations have experienced a reduction in STR (short tandem repeat) diversity, heterozygosity across STR loci being reduced from approximately 0.7 to 0.6, compared to cosmopolitan, non-African populations [37]. Although several rCNVs overlap genomic regions known to harbor structural variation, comparison with global reference data from gnomAD-SV indicates that many of these rCNVs are rare or absent in global populations but occur at elevated frequencies in the Native American cohorts. The higher prevalence of rCNVs, including rCNVs of shared origin, is consistent with the founder nature of these populations. Alternatively, the rCNVs could be maintained by balanced selection, although this has not been demonstrated. It remains unknown whether the high frequency and persistence of rCNVs in Native American populations is due to neutral drift, founder effects, or selection; probably, each of these possibilities remains viable given the available data.

Both population isolates and families represent sampling frameworks in which private alleles are more likely to be observed [38], and studies of genetic diseases in these contexts have led to many discoveries in medical genetics. The rCNVs we observed in Native Americans are not private mutations in the sense that they are not limited to individual families, or even a single tribe. Instead, these rCNVs were widely distributed, with several being abundant in both tribes. To test familial clustering, we compared the average coefficient of relationship of rCNV carriers to the overall coefficients of relationship of the tribes (Figures S1–S2). Consistent with these rCNVs having persisted for many generations, the coefficients of relationship among the carriers did not differ from the overall coefficients of relationship in the tribes. Within available pedigrees, rCNVs were observed to be transmitted across relatives in patterns consistent with Mendelian inheritance. Although related carriers were observed, the distribution of kinship coefficients among carriers paralleled that of the full cohort, consistent with these variants occurring across these populations rather than being confined to a single lineage. In some cases, the same rCNV was observed in both tribes, further supporting their persistence over many generations.

The persistence of certain rCNVs for hundreds or even thousands of generations may seem paradoxical given potential deleterious effects. Such long-term persistence could reflect incomplete penetrance, drift in small founder populations, or other evolutionary mechanisms that allow CNVs to remain in populations despite modest phenotypic effects.

This study was not structured to identify de novo CNVs or to capture their effects; parent–child trios are optimal for such analyses. Nevertheless, de novo CNVs are well known to contribute substantially to neuropsychiatric disease risk, accounting for up to 25% of the CNV burden associated with autism, approximately one-third of all autism cases, and up to two-thirds of cases arising in low-risk families [39]. In Schizophrenia, de novo CNVs also play an important role [40], the genes disrupted being enriched for development and synaptic function. Relative to genes implicated in schizophrenia via GWAS, these CNVs confer a high level of risk, with ORs ranging from 3 to 30. Thus, they should be subject to strong negative selection [2, 40, 41] due to their contributions to schizophrenia, and as pleiotropic risk factors in cognitive disability, epilepsy, and autism as well, all being phenotypes that can directly or indirectly lead to decreased fertility [40]. Thus, CNVs associated with diseases causing decreased reproductive fitness must be constantly replenished in populations by de novo mutation.

Correspondingly, as a group, de novo CNVs are more likely to be deleterious than CNVs transmitted through several generations. Although some of these rCNVs identified here overlap genes previously implicated in psychiatric disorders (e.g., *PRODH* within 22q11.2 region) [42], associations with AUD or “Any psychiatric disorder” did not reach statistical significance after correction for multiple testing, likely reflecting limited sample size. Importantly, odds ratios remained elevated after accounting for relatedness and demographic covariates (AUD OR 3.16–3.19; psychiatric OR 4.69–4.80) although confidence intervals were wide. These findings should therefore be interpreted as exploratory and hypothesis generating rather than definitive evidence of pathogenicity. Using transcriptomic analysis, we found gene network changes representing *trans* effects on expression of other genes in carriers of the 6p21.33 rCNV. While the *cis* dosage effects on *MICA* and *HCG-26* were robust and expected, the observed *trans* effects are based on a small number of LCLs and may be influenced by technical variability; therefore, they should also be interpreted with caution. Functional consequence of the 6p21.33 rCNV would be consistent with the evolutionary conservation of genes within the rCNV (e.g., *MICA*, *HCP5*, *PMSP*, *HCG26*) and with phenotypic consequences reported in mouse models when orthologous MHC class I-related loci are disrupted [43], further reinforcing the idea that rCNVs deleting or duplicating genes are likely to alter phenotype, and fitness even if they do not have catastrophic effects. However, an important limitation of this study is the lack of a comprehensive set of physiological and biochemical measures that might have captured non-psychiatric or more subtle effects of rCNVs and using binary diagnostic phenotypes may obscure more subtle genotype phenotype associations.

An additional limitation of this study is diagnostic heterogeneity across cohorts, including differences in diagnostic instruments (SADS-LA versus SCID) and DSM versions (DSM-III-R versus DSM-5). While AUD represents a relatively stable diagnostic construct across DSM iterations and was therefore prioritized as the primary outcome, the composite outcome labeled “Any psychiatric disorder” aggregates non-equivalent diagnoses across cohorts. Variation in instruments and criteria may therefore obscure gene effects and reduce power.

On an individual basis, the 22q11.2 rCNV associated with AUD and “Any psychiatric disorder” showed elevated odds ratios, although these estimates are based on a limited number of carriers and should be interpreted cautiously. These results, while not significant after correction for multiple testing, illustrate the potential impact of specific rCNVs on psychiatric phenotypes. The ability to link a specific rCNV to AUD or other discrete disease phenotype is limited by sample size. The associations of the rCNV duplication of the Chr 22 VCF region to “Any psychiatric disorder” and AUD are therefore instructive since they are based on 27 rCNV carriers, 22 of whom had AUD, compared to the expected 16 based on the overall prevalence of AUD in this sample of PI. Furthermore, 24 out of the observed 27 heterozygotes had a psychiatric disorder. Thus, these ORs are far larger than for a typical GWAS finding in psychiatric disease, although the confidence intervals are wide, reflecting uncertainty in the estimates. The magnitude of these effects would be comparable to the effects of the functional *ALDH2* and *ADH1B* variants on AUD and alcohol drinking although the confidence intervals are wide and reflect uncertainty in the estimates. This suggests that other rCNVs and CNVs might alter risk for AUD but with lower OR or lower abundances that would render their effects undetectable in samples of the size we studied. We limited our current analysis to rCNVs with frequencies > 2.2% such that a large effect on risk (OR = 10) could be detectable with 80% power, and via this approach, we would also be able to detect effects with smaller ORs, albeit with reduced power.

The clinical manifestations of 22q11.21 deletions causing haploinsufficiency of 30–50 genes are diverse, ranging from cardiac anomalies, facial dysmorphism, developmental delay, and schizophrenia. Collectively, 22q11.21 deletion CNVs are common, occurring in approximately 1:3000–1:4000 live births. However, smaller 22q11.21 duplications are even more common, representing 1:1600 live births in Denmark [44]. These 22q11.21 duplications have been implicated in autism spectrum disorder, intellectual disability, and bipolar disorder, with the penetrance altered by the size of the duplicated segment and the genes duplicated [45]. Together with variations in genetic background and environmental exposure, it is understandable that the structural diversity of 22q11.21 CNVs leads to phenotypic differences from individual to individual and family to family, and this can be taken as an observation likely to apply at least in part to other CNVs that duplicate or delete genes in a variable fashion.

The 22q11.21 rCNV associated with “Any psychiatric disorder” in this study duplicates genes plausibly altering brain function: *USP18*, *DGCR6*, and *PRODH*. *USP18* (Ubiquitin Specific Peptidase 18), a deubiquitinase, play a critical regulatory role in immune response, particularly type I interferon (IFN-I) signaling. Dysregulation of *USP18* has been implicated in a spectrum of diseases, including autoimmune disorders, cancer, and neurological conditions [46]. *DGCR6* (DiGeorge Syndrome Critical Region Gene 6) exerts a regulatory role on neural crest cell migration and early neurodevelopment. *DGCR6* apparently modulates the expression of neighboring genes such as *TBX1* (T-Box Transcription Factor 1). *DGCR6* variants have been associated with schizophrenia susceptibility and brain connectivity, suggesting that duplications of this gene might have downstream effects on behavioral regulation. *PRODH* (Proline Dehydrogenase 1) encodes a key enzyme crucial for proline catabolism that impacts glutamatergic neurotransmission. Whilst *PRODH* genetic variants have been linked to impulsivity, cognitive deficits, and altered prefrontal cortex activity, increased gene dosage due to *PRODH* duplications perturbs proline-to-glutamate conversion, potentially disrupting the excitatory/inhibitory balance in key brain regions implicated in addiction vulnerability [47].

As previously mentioned, rCNVs may exert stronger effects on non-psychiatric phenotypes not analyzed in this study. The 20p12.1 rCNV found in both Native American populations deletes *MACROD2*, a deacetylase that specifically targets the removal of ADP-ribose from mono-ADP-ribosylated proteins. Mutations or dysregulation of *MACROD2* have been associated with several diseases, including neurodevelopmental disorders such as ASD and ID [48]. Notably, the *MACROD2* rCNV had an OR of 4.9 for AUD (95% CI 1.1–22.1), potentially indicating a large effect although this estimate should be interpreted cautiously given limited sample size. The presence of rCNVs in Native Americans highlights the importance of expanding genomic studies to diverse populations. Native Americans and other well-defined populations carry alleles contributing to their unique characteristics and frequency of private alleles, while examples from other populations, such as Finnish [49] or South Asians (gnomad) where *HTR2B* stop codons, exist. Native Americans similarly are likely to harbor population-specific variants that may influence phenotype and disease susceptibility. Whilst many population-specific variants are thought to be non-pathogenic, some functional polymorphisms are maintained in particular populations by selection for diverse phenotypes and for example resistance to malaria [50] dietary lactose [51], and more speculatively, plague [52].

It is unknown what proportion of heritability of AUD and other psychiatric phenotypes that ongoing expansion in size of GWAS will eventually capture, GWAS being performed with common SNPs either directly genotyped or imputed [53]. Notably, certain CNVs in schizophrenia, such as 22q11.2 and 3q29 deletion, confer substantial individual risk with ORs ranging from 20 to 40. While these CNVs were not observed in our Native American cohorts, it is useful context that other loci such as 16p11.2 duplications, 1q21.1 deletions, and *NRXN1* deletions exhibit more moderate effects with ORs ranging from 5 to 12 [2, 15, 54, 55]. In contrast, the genetic liability conferred by rare CNVs in ASD has been estimated to account for 5–10% of cases, particularly through de novo events [56, 57]. Other sources of genetic variance that are largely untapped by GWAS are rare SNVs and STR (short tandem repeat) polymorphisms whose genotypes are not readily captured by proxy SNPs. Prospectively, study of these additional types of genetic variation, and in contexts such as founder populations, may lead to the discovery of additional loci of large effect.

## Conclusions

The genetic risk for AUD and related psychiatric disorders arises from a complex interplay of common and rare variants, most of which remain unknown. The enrichment of rCNVs in founder populations such as Native Americans suggests that linkage of CNVs to psychiatric diagnoses might also be detectable in families. Our analysis, and others, show the *cis* and *trans* molecular consequences of CNVs that delete and duplicate genes. This has important implications for future studies in large and ancestrally diverse datasets where it will be possible to aggregate non-recurrent CNVs to deepen our understanding of psychiatric disease etiology. By moving beyond traditional GWAS to incorporate structural variants, future studies may uncover biological mechanisms that have so far remained hidden.

## Methods

### Subjects

We studied 395 members of a PI tribe in Oklahoma, and 370 members of a SWI tribe in Arizona, the names of the tribes being withheld. In both, participants were collected as part of AUD research on super pedigrees selected based on structure and accessibility rather than relationship to an affected proband. In these Native American communities, AUD is common, and thus, it was unnecessary to select pedigrees based on phenotype or relationship to an affected proband, and this was therefore not done. KING-robust kinship coefficients were computed in PLINK2 using the KING algorithm, after exclusion of SNPs with a missing rate > 0.1 and minor allele frequency < 0.01 [58]. The coefficients of relationship of the study participants were equivalent to those of the overall populations of these two tribes, and just below the second cousin level, as previously reported. A descriptive analysis of the pairwise kinship coefficients shows that while the overall populations are largely unrelated (Figure S1), carriers of certain rCNVs as seen in our representative examples in Figure S2 are substantially more related to each other than expected by chance. This is consistent with co-segregation within these extended super-pedigrees. To ensure this clear pattern of familial relatedness did not confound our findings, all association analyses were corrected using pairwise kinship coefficients within a generalized linear mixed model (GLMM) framework, kinship having been computed based on SNP array genotypes. To determine the minimum rCNV frequency detectable with adequate power, we performed a power calculation assuming a two-sided *α* = 0.05 and 80% power. Based on this, our primary analyses focused on rCNVs present at ≥ 2.2% frequency which could be detected with adequate power to observe large effect sizes (OR ≈10). For comparison, we studied 1458 unrelated individuals representing a cosmopolitan case/control AUD sample at the NIH CC, these patients also having high frequencies of other SUDs and other psychiatric disorders. The NIH CC cohort is genetically heterogeneous, as reflected by its broad distribution in a multidimensional scaling (MDS) plot based on pairwise identity-by-state genetic distances from genome-wide SNP data (Figure S7), with points colored by self-reported race. The cohort was treated as a cosmopolitan comparison group.

All Native American participants underwent assessment using the Structured Assessment for Diagnosis–Lifetime Version (SADS-LA) and the Structured Clinical Interview for DSM (SCID) was used for the NIH CC sample. Psychiatric diagnoses were assigned by consensus conference and via DSMIII-R criteria (PI and SWI) or DSM-5 criteria (NIH CC). In addition, the NIH CC sample was genotyped on a different Illumina array than the Native Americans consequently; CNV comparisons are presented for general benchmarking rather than as fully integrated with the primary analyses of the Native American samples. Because diagnostic instruments and DSM criteria differed across cohorts, analyses of “Any psychiatric disorder” were intended as broad measures of psychiatric burden rather than harmonized diagnostic entities. All participants were studied under NIH IRB-approved human research protocols and provided informed consent in accordance with the Declaration of Helsinki, including consent for genomic studies, and as approved by the Tribal Councils of both tribes, thus also representing group consent.

### Genotyping

Genomic DNA was prepared from LCLs (Native Americans) or venous blood (NIH CC sample). Two Illumina genotyping arrays were used: for PI, the Infinium HumanHap 550 array, which includes 561,466 SNPs of which 98,656 are located in regions of known CNVs; and for SWI and NIH CC, the Infinium Human OmniExpress Exome array, which includes 962,215 SNPs, of which 206,665 are located in regions of known CNVs [59]. Samples with an overall call rate of < 98% were excluded. This resulted in the exclusion of 48 samples such that the final sample sizes were PI 387, SWI 350, and NIH CC 1438. The genotype reproducibility rate was 0.999 based on 91 duplicate sample pairs. For CNV calling, log R ratio (LRR) and B allele frequency (BAF) data were exported from normalized Illumina datasets using the CNV Partition tool in Genome Studio (Illumina). CNV detection was independently performed using PennCNV (version 1.0.3), which applies a Hidden Markov model (HMM) to integrate LRR, BAF, and SNP allele frequencies. Shared CNVs were defined as those detected by both methods with ≥ 50% reciprocal overlap of inferred breakpoints, and only these shared CNVs were retained for downstream analyses. To minimize potential platform or batch-specific artifacts, CNVs were retained only if they spanned at least ten adjacent SNPs and were ≥ 10 kb in size. All CNV calls were manually reviewed by inspecting LRR and BAF plots and only autosomal SNPs with known positions were considered. CNV annotation was performed using the hg19 (GRCh37) reference genome. rCNVs were initially identified based on overlapping inferred breakpoints and then confirmed by haplotype analysis, with all candidate rCNVs occurring on the same haplotype backgrounds in the 3’ and 5’ regions flanking the CNV. Although exact CNV breakpoints are approximate due to array resolution (e.g., within approximately 10 kb), haplotype sharing across carriers increased confidence in rCNV identification. Global population frequencies for rCNV loci were assessed using gnomAD-SV v2.1 to provide population level context. For rCNVs overlapping multiple gnomAD-SV variants, global frequencies were interpreted as ranges reflecting breakpoint heterogeneity rather than as single representative allele frequencies.

### Haplotype and Coalescence Analyses

To evaluate haplotype background, genotypes of SNPs flanking candidate rCNVs were examined utilizing Haploview 4.2 [60]. The number of generations since each rCNV was introduced into the population by mutation or introgression was estimated using the Gamma method with an assumption of independence [61]. The coalescence time of chromosomes with and without the allele was inferred based on the assumption that the shared haplotype of a variant, representing the distance to the first recombination site, decreases with an increasing number of generations. Among individuals who were not first- or second-degree relatives, 60 SNPs were selected both upstream and downstream of each rCNV and phased using Beagle 5.4 [62, 63]. Shared haplotypes were successively extended via SNPs yielding congruent haplotypes or if the alleles at the five successive SNPs matched, thus “rescuing” point mutations and the rare genotyping error. The parameter *τ* was estimated by 2/${l}_{ave}$, where ${l}_{ave}$ is the weighted average of maximum shared haplotype sizes. The weights were derived from the total numbers of haplotypes that had at least one common breakpoint defining a maximum shared haplotype region. The sex-averaged local recombination rates were used, replacing the uniform recombination rate assumed in the Haldane model [64]. Confidence intervals were calculated for all coalescence time estimates to reflect uncertainty arising from variation in shared haplotype length and local recombination rates.

### Differential Expression Analysis

To detect *cis* and *trans* effects of a common rCNV on the transcriptome, we compared gene expression in lymphoblastoid cell lines from five individuals with a 6p21.33 rCNV deletion against four individuals lacking the CNV, and we also evaluated *cis* effects of other CNV deletions and duplications in these nine LCL transcriptomes. Briefly, ~ 10 μg total RNA was reverse transcribed using the Superscript Vilo cDNA Synthesis Kit (Thermo Fisher Scientific, Waltham, MA) and the cDNA used for the Ion AmpliSeq™ Transcriptome Human Gene Expression Core Panel Kit (Thermo Fisher Scientific, Waltham, MA). Sequencing was performed using Ion 550 chips on an Ion S5 Sequencer (Thermo Fisher Scientific, Waltham, MA) yielding approximately 20 million reads per sample, with an average length of 115 bp. Reads were mapped to the hg19 genome reference in Torrent Suite™ Software 5.18.2. The differential gene expression was analyzed in R using DESeq2. Ingenuity pathway analysis (IPA) was used to identify canonical pathways and gene networks.

### Association Analyses

Associations of individual rCNVs observed in sufficient numbers for power to detect an effect of large size (see the “Results” section) were initially evaluated by c2 test with Bonferroni adjustment, all expected cell sizes being > 5. Multiple testing was accounted for by applying Bonferroni correction within each rCNV group. The primary phenotypes tested were AUD versus no AUD and “no psychiatric diagnosis” versus “Any psychiatric diagnosis” (including AUD, phobia, MDD, PTSD, obsessive compulsive disorder (OCD), SUD, antisocial personality disorder (ASPD), generalized anxiety disorder, schizophrenia, and bipolar disorder). All association analyses for the rCNVs were performed using generalized linear mixed models (GLMMs) including pairwise kinship coefficients as a random effect and age and sex as fixed-effect covariates to account for relatedness and demographic confounding.

### Burden Analyses

CNV burden was measured in two ways: number of CNVs and number of genes deleted or duplicated by CNVs. CNV burden was compared between cases and controls nonparametrically (Mann–Whitney Wilcoxon) for AUD and “Any psychiatric disease.” These analyses were performed without adjustment for covariates such as age, sex, or ancestry, and without stratification by CNV type, size, or gene constraint. Burden analyses were intended as descriptive and exploratory and were not used for primary statistical inference.

## Supplementary Information

Below is the link to the electronic supplementary material.

ESM 1 (DOCX 1.37 MB) 

## Data Availability

The data supporting the analyses and findings of this study are available on request from the corresponding author.

## Abbreviations

ADHD: Attention deficit hyperactivity disorder
ASPD: Antisocial personality disorder
AUD: Alcohol use disorder
BAF: B allele frequency
CNVs: Copy number variants
GLMM: Generalized linear mixed model
GSTM1: Glutathione S-transferase
gnomAD-SV: Genome Aggregation Database – Structural Variation
GWAS: Genome-wide association studies
HMM: Hidden Markov model
ID: Intellectual disability
IPA: Ingenuity pathway analysis
LCLs: Lymphoblastoid cell lines
LRR: Log R ratio
MDD: Major depressive disorder
NAHR: Nonallelic homologous recombination
NIH CC: NIH Clinical Center
OCD: Obsessive compulsive disorder
OR: Odds ratio
PI: Plains Indians
PTSD: Post-traumatic stress disorder
rCNV: Recurrent copy number variant
SADS-LA: Structured Assessment for Diagnosis–Lifetime Version
SCID: Structured clinical interview for DSM
SNPs: Single nucleotide polymorphisms
STR: Short tandem repeat
SUDs: Substance use disorders
SWI: Southwest Indians
VCF: Velocardiofacial syndrome

## Declarations

### Disclaimer

This research was supported by the Intramural Research Program of the National Institutes of Health (NIH). The contributions of the NIH author(s) are considered works of the United States Government. The findings and conclusions presented in this paper are those of the authors and do not necessarily reflect the views of the NIH or the US Department of Health and Human Services.
