# Supplementary material for: Recurrent and Non-Recurrent Copy Number Variants in Native Americans and a Cosmopolitan Sample in Relation to Alcohol Use Disorder and Other Psychiatric Diseases
Source: Mol Neurobiol. 2026 Mar 23;63(1):515. doi: 10.1007/s12035-026-05808-w (PMC13006474; doi:10.1007/s12035-026-05808-w)
Supplement: Supplementary file 1 — (DOCX 1.37 MB) [file 12035_2026_5808_MOESM1_ESM.docx]

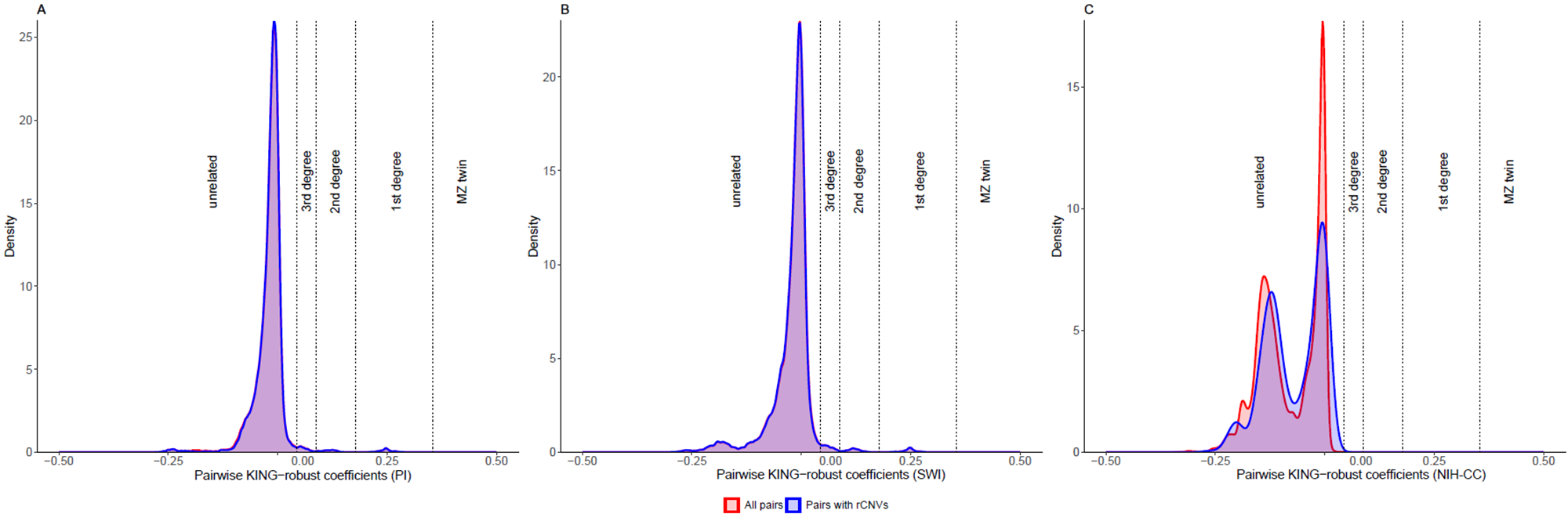


**Figure S1. Pairwise kinship coefficients for three populations comparing pairs of individuals in which both have at least one rCNV versus all pairs**

Density plots of KING-robust kinship coefficients for all pairs of individuals in (A) Plains Indians (PI), (B) Southwest Indians (SWI) and (C) NIH Clinical Center (NIH CC) sample, there being 74,691 pairs for PI, 61,075 pairs for SWI and 1,033,203 pairs for the NIH CC sample. Blue/purple represents pairs with at least one rCNV, while red/purple indicates all pairs, purple being indicative of the area of overlap. Vertical dashed lines denote thresholds for kinship categories: unrelated, 3rd degree, 2nd degree, 1st degree, and monozygotic (MZ).


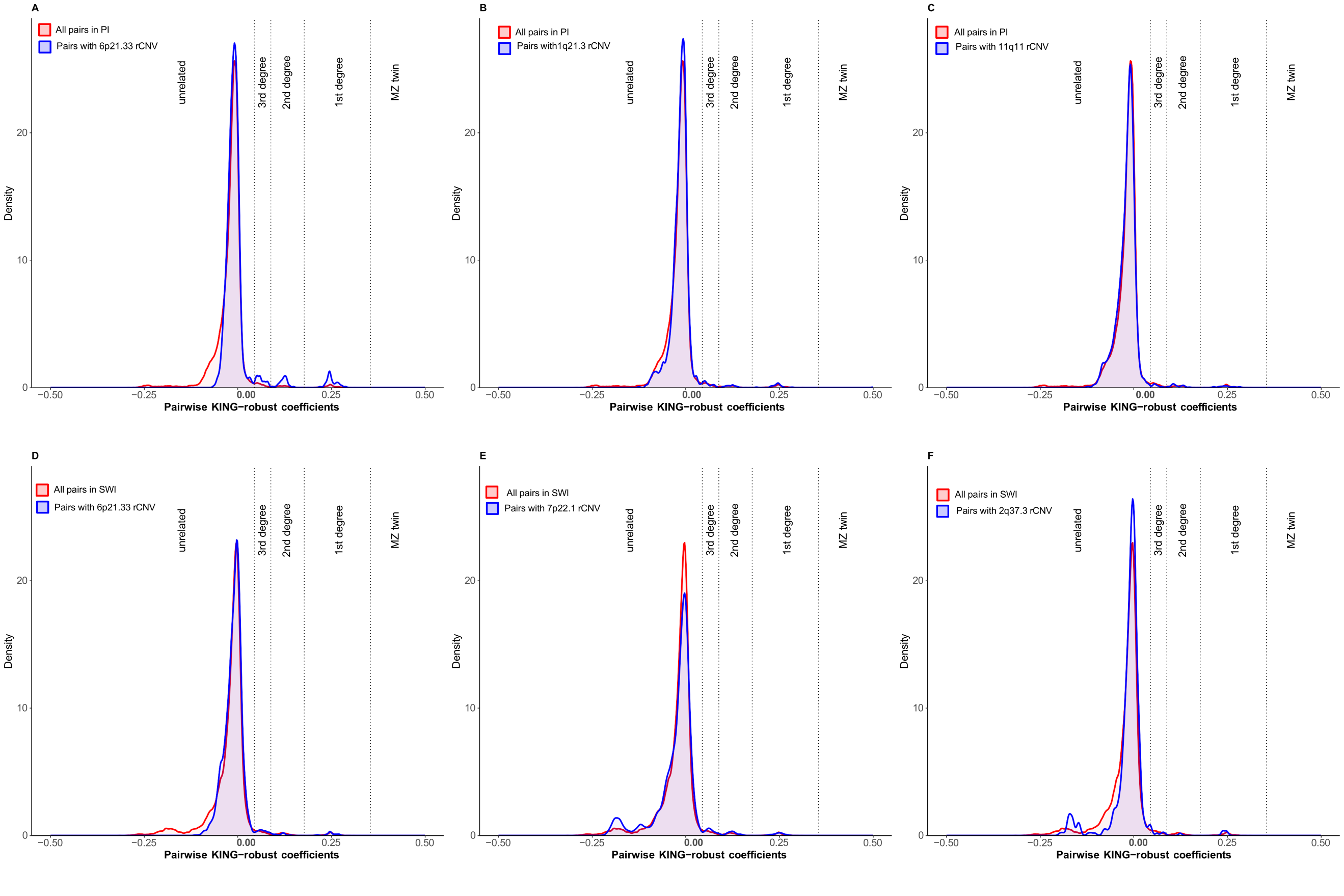


**Figure S2. Distribution of pairwise kinship coefficients among carriers of selected high-frequency rCNVs in PI and SWI Native American populations** **versus all pairs****.**

Density plots of KING-robust kinship coefficients for pairs of individuals carrying selected high-frequency rCNVs in Plains Indians (PI; top) and Southwest Indians (SWI; bottom). The left panels represent the shared rCNV at 6p21.33. The middle and right panels represent the most frequent population-specific rCNVs in each cohort (PI: 1q21.3 and 11q11; SWI: 7p22.1 and 2q37.3). In each panel, the blue/purple density histogram represents pairs in which both individuals carry the rCNV, and the red/purple histogram represents all pairs in the sample (74,691 for PI and 61,075 for SWI), with purple indicating overlap. Vertical dashed lines denote thresholds for kinship categories: unrelated, 3rd degree, 2nd degree, 1st degree, and monozygotic (MZ).


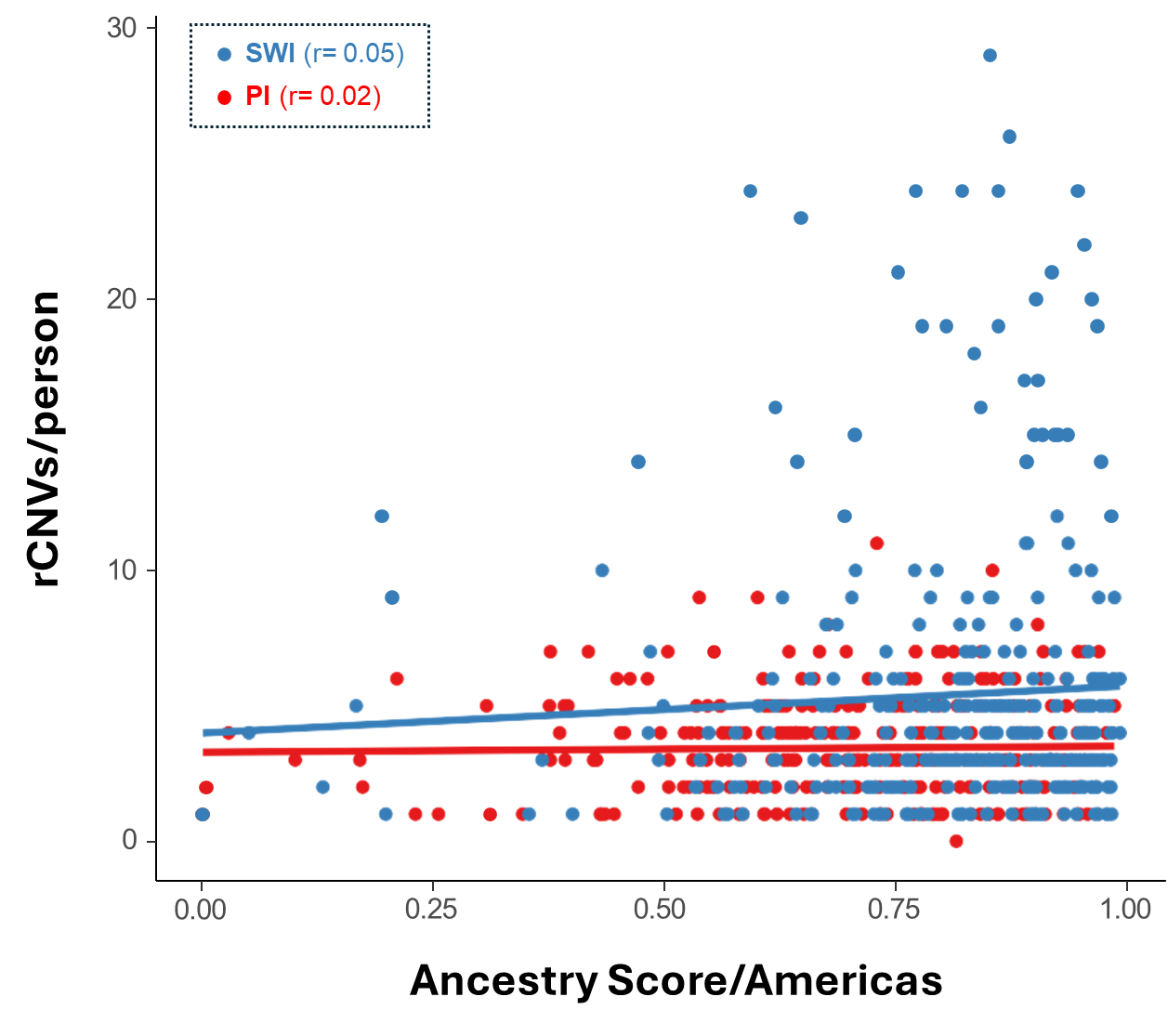


**Figure S3.** **rCNV number per individual vs Americas ancestry**

Americas ancestry score estimated via Ancestry Informative Markers (AIMs) in SWI and PI. Pearson correlations showed a lack of relationship between admixture and number of rCNVs/person.


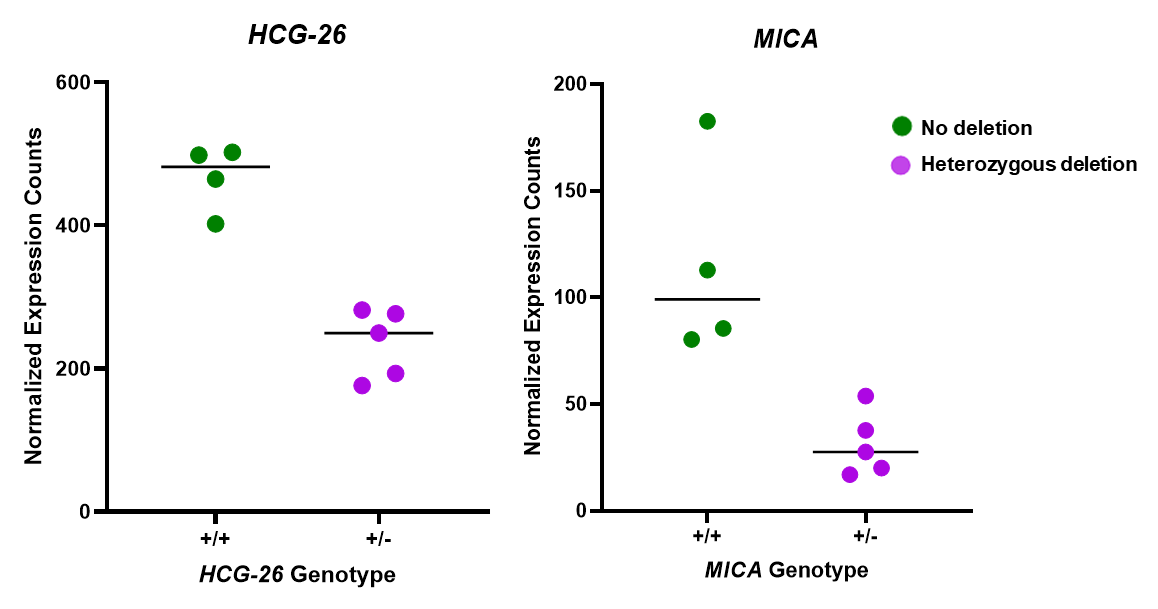


**Figure S4.** **Expression profiles of genes deleted by the 6p21.33 rCNV**

The expression profiles of *MICA* and *HCG-26*, two genes deleted by the 6p21.33 rCNV, in lymphoblastoid cell lines from individuals without the deletion and heterozygous for this rCNV, and putatively representing *cis* effects of this rCNV.

**
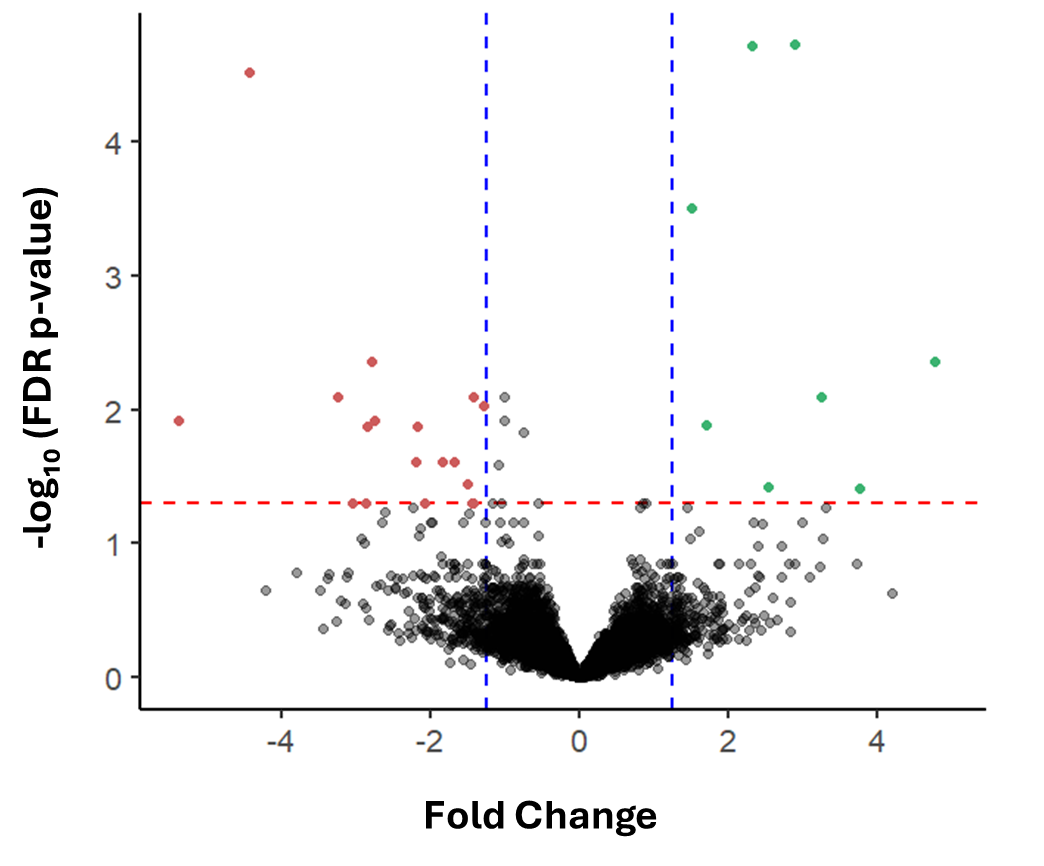
**

**Figure S5.** ***Trans* effects on transcriptome in 6p21.33 rCNV deletion carriers versus non-carriers**

Volcano plot showing differential expression of genes in LCLs from Plains Indian**s** driven by the 6p21.33 rCNV deletion and predominantly representing *trans* effects of this rCNV. Red and green dots indicate significantly upregulated and downregulated genes, respectively (|fold change| >1.25; FDR < 0.05). The red dashed horizontal line represents a false discovery rate (FDR) threshold of 0.05 and the blue vertical lines represent the 1.25-fold change cutoffs.


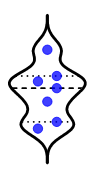

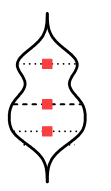


**0**

**1**

**2**

**3**

**Duplications**

**Deletions**

**Relative Expression (Normalized Counts)**

**Figure S6.** **The normalized relative expression of genes putatively affected in *cis* by heterozygous (rCNVs) in lymphoblastoid cell lines (LCLs).**

Blue circles represent duplications; red squares represent deletions. Each point corresponds to a unique gene-CNV combination. Expression levels were normalized to the mean (= 1) of non-CNV homozygotes.

**
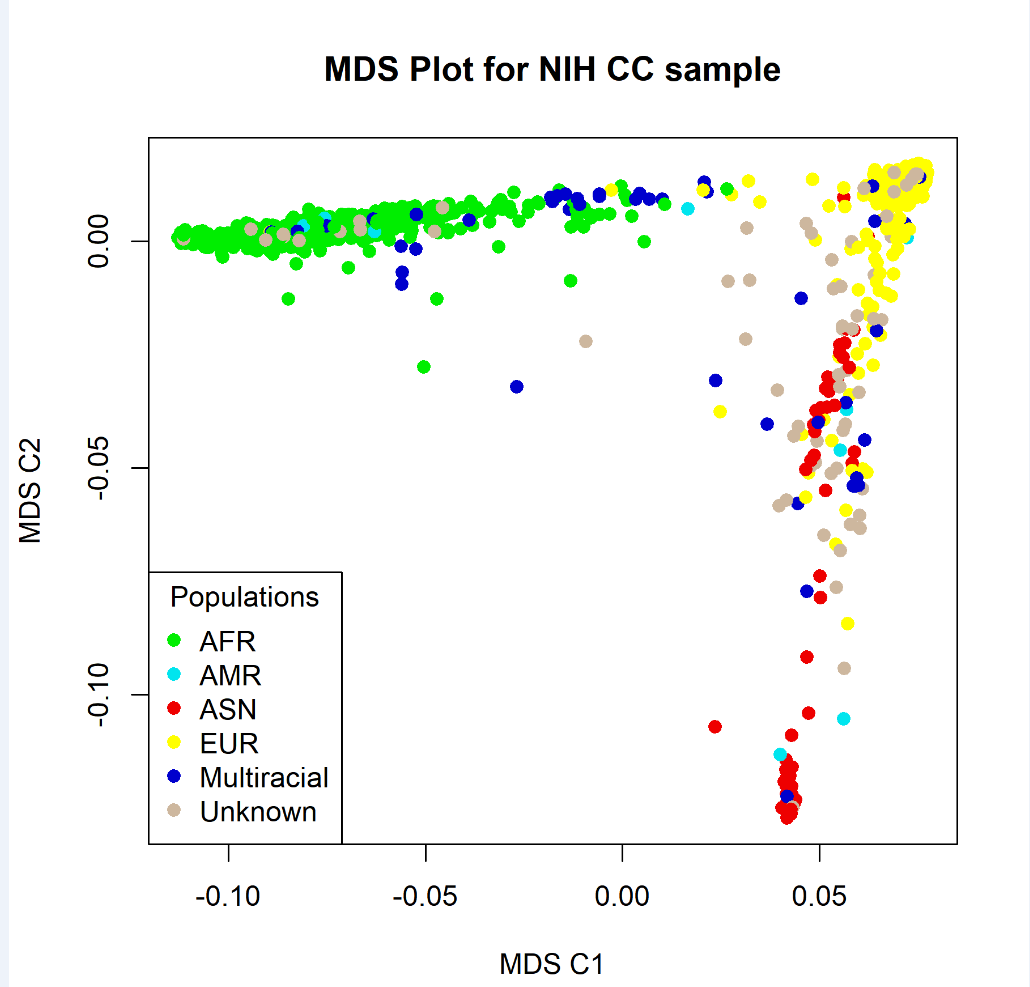
Figure S7. Multidimensional scaling (MDS) plot of the NIH CC cohort showing measured ancestry.**

Ancestry was computed from genome-wide SNP data using PLINK. The first two MDS components (MDS C1 and MDS C2), captured the major axes of genetic variation among individuals and were used for visualization. Each point represents one individual, , and points are color-coded based on self-reported race.

**
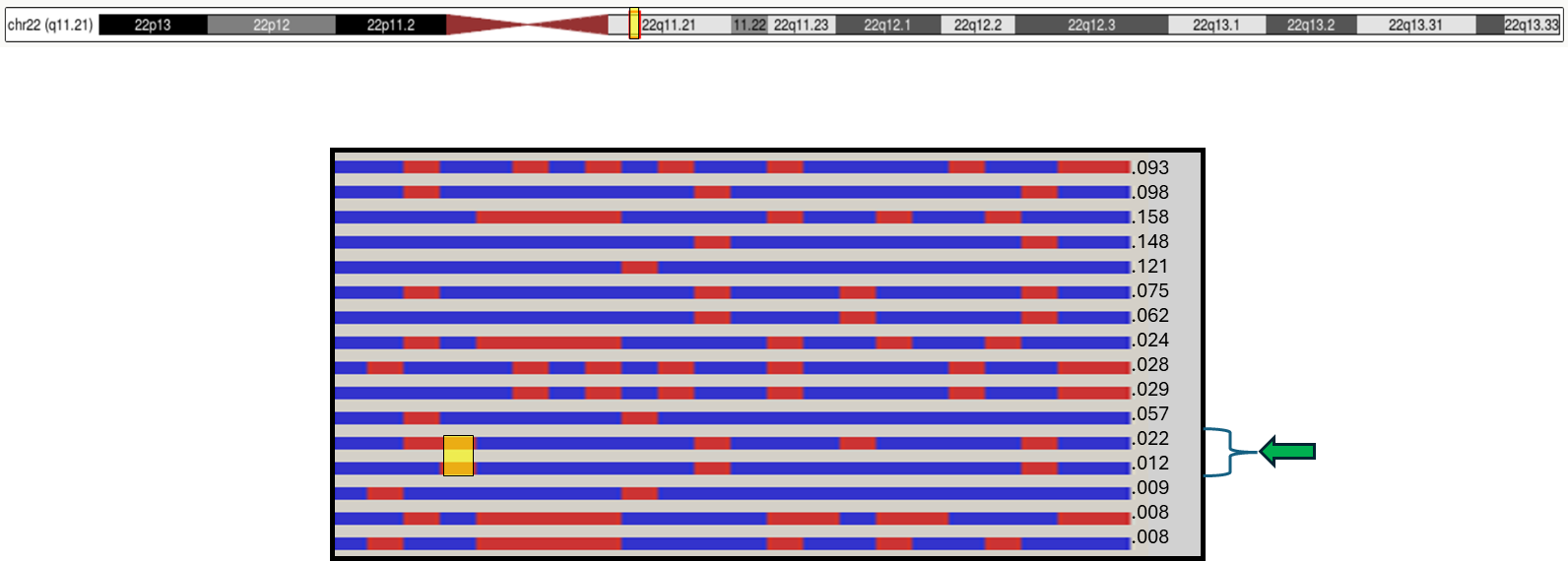
**

**Figure S8. Distinct haplotypes shared by 22q11.21 rCNV chromosomes in Plains Indians (N=387).**

This figure shows distinct shared haplotypes of the carriers of the 22q11.21 rCNV. The yellow box denotes the location of the 22q11.21 duplication, and the green arrow indicates a haplotype (frequency 0.022) and a daughter haplotype (frequency 0.012) containing the 22q11.21 rCNV.

|  | **Plains Indians** | | | **Southwest Indians** | | | **NIH CC** | | |  | |
| --- | --- | --- | --- | --- | --- | --- | --- | --- | --- | --- | --- |
| **Phenotype** | **Frequency** | **CNV Load (Mean ± SD)** | **Gene Load (Mean ± SD)** | **Frequency** | **CNV Load (Mean ± SD)** | **Gene Load (Mean ± SD)** | **Frequency*** | **CNV Load (Mean ± SD)** | **Gene Load (Mean ± SD)** |  |  |
| AUD | 224 (0.60) | 3.6 ± 2.1 | 12.8 ± 74.4 | 233 (0.71) | 5.1 ± 5.2 | 17.4 ± 39.0 | 853 (0.62) | 2.9 ± 2.3 | 12.8 ± 22.5 |  |  |
| Non-AUD | 151 (0.40) | 3.5 ± 2.2 | 13.8 ± 60.0 | 99 (0.29) | 4.8 ± 3.9 | 10.6 ± 20.4 | 521 (0.38) | 2.8 ± 2.2 | 11.5 ± 14.9 |  |  |
| p-value |  | 0.3 | 0.8 |  | 0.1 | 0.5 |  | 0.9 | 0.8 |  |  |
| Phobia | 41 (0.11) | 3.6 ± 1.6 | 7.6 ± 12.0 | 89 (0.27) | 4.8 ± 4.8 | 14.1 ± 24.6 | 145 (0.11) | 3.0 ± 2.4 | 10.8 ± 11.1 |  |  |
| Non-Phobia | 334 (0.89) | 3.5 ± 2.2 | 14.0 ± 73.0 | 243 (0.73) | 4.9 ± 4.8 | 15.9 ± 37.6 | 1131 (0.89) | 2.9 ± 2.2 | 12.6 ± 21.1 |  |  |
| p-value |  | 0.4 | 0.7 |  | 0.6 | 0.1 |  | 0.9 | 0.9 |  |  |
| MDD | 27 (0.07) | 3.7 ± 2.1 | 5.8 ± 4.7 | 35 (0.11) | 5.1 ± 5.0 | 25.4 ± 62.0 | 250 (0.20) | 2.9 ± 2.3 | 12.0 ± 18.2 |  |  |
| Non-MDD | 348 (0.93) | 3.6 ± 2.2 | 13.7 ± 71.3 | 297 (0.89) | 4.9 ± 4.8 | 14.2 ± 30.0 | 1027 (0.80) | 2.9 ± 2.2 | 12.8 ± 21.0 |  |  |
| p-value |  | 0.5 | 0.5 |  | 0.9 | 0.5 |  | 0.1 | 0.07 |  |  |
| PTSD | 17 (0.04) | 4.2 ± 3.8 | 39.5 ± 142.7 | 38 (0.11) | 7.2 ± 7.6 | 26.9 ± 46.9 | 167 (0.13) | 2.8 ± 2.3 | 13.1 ± 33.1 |  |  |
| Non-PTSD | 353 (0.96) | 3.5 ± 2.0 | 12.5 ± 64.7 | 294 (0.89) | 4.7 ± 4.3 | 13.9 ± 32.5 | 1101 (0.87) | 3.0 ± 2.3 | 12.3 ± 17.6 |  |  |
| p-value |  | 0.68 | 0.51 | 0.08 | 0.1 | 0.2 |  | 0.2 | 0.1 |  |  |
| OCD | 4 (0.01) | 3.8 ± 1.2 | 6.0 ± 2.2 | 9 (0.03) | 4.0 ± 4.1 | 12.8 ± 19.4 | 13 (0.01) | 2.8 ± 1.4 | 9.7 ± 7.7 |  |  |
| Non-OCD | 371 (0.99) | 3.5 ± 2.2 | 13.3 ± 69.1 | 323 (0.97) | 5.0 ± 4.8 | 15.3 ± 34.5 | 1262 (0.99) | 2.9 ± 2.3 | 12.5 ± 20.3 |  |  |
| p-value |  | 0.8 | 0.9 |  | 0.3 | 0.4 |  | 0.6 | 0.9 |  |  |
| ASPD | 46 (0.12) | 3.3 ± 1.8 | 4.5 ± 5.4 | 90 (0.27) | 4.5 ± 4.6 | 11.2 ± 22.7 |  |  |  |  |  |
| Non-ASPD | 329 (0.88) | 3.6 ± 2.1 | 14.4 ± 73.3 | 242 (0.73) | 5.1 ± 4.9 | 17.1 ± 38.2 | __ | __ | __ |  |  |
| p-value |  | 0.3 | 0.01 |  | 0.09 | 0.2 |  |  |  |  |  |
| SUD | 42 (0.11) | 3.9 ± 1.7 | 28.6 ± 145.4 | 29 (0.09) | 5.0 ± 4.9 | 18.7 ± 47.7 | 531 (0.39) | 2.8 ± 2.3 | 12.6 ± 25.0 |  |  |
| Non-SUD | 333 (0.89) | 3.5 ± 2.2 | 14.0 ± 72.8 | 303 (0.91) | 4.9 ± 4.8 | 15.1 ± 33.1 | 843 (0.61) | 2.9 ± 2.2 | 11.7 ± 15.4 |  |  |
| p-value |  | 0.07 | 0.4 |  | 0.7 | 0.4 |  | 0.02 | 0.1 |  |  |

**T****able S1**: Relationship of CNV burden (measured by number of CNVs or Genes) to “Any Psychiatric Disorders” or AUD. (Mann-Whitney Wilcoxon).

*For the various diagnoses the number of individuals represented differ due to missingness of data.

| **Cytogenetic region** | **CNV type** | **Size (kb)** | **Allele Frequencies** | | **Genes Affected** | **Estimated Generations (95% CI)** | **Estimated Age in Years** | **Phenotype** |
| --- | --- | --- | --- | --- | --- | --- | --- | --- |
|  |  |  | **SWI** | **PI** |  |  |  |  |
| 6p21.33 | Deletion | 98 | 0.14 | 0.08 | *MICA, HCP5, PMSP, HCG26* | 1,405  (988-1925) | 28,100 | Located within the MHC region, *MICA* affects immune signalling, with roles in inflammation & autoimmunity. Deletions in the MHC region have been associated with psoriasis and other autoimmune diseases. |
| 13q13.1 | Deletion | 55 | 0.07 | 0.1 | *Non-genic region (near SLITRK1)* | 813  (623-1040) | 16,270 | *SLITRK1* is a brain-expressed gene involved in neuronal development and synaptic connectivity. *SLITRK1* is associated with Tourette syndrome, OCD and ADHD. |
| 3p25.2 | Duplication | 177 | 0.02 | 0.05 | *RAF1, TMEM40* | 1,415  (954-2020) | 28,100 | *RAF1* encodes a MAPK pathway kinase regulating cell proliferation and survival. *RAF1* CNVs are associated with cardiovascular disease and cancer. *TMEM40*,is a transmembrane protein associated with cancer |
| 22q11.23- q21.1 | Duplication | 260 | 0.11 | 0.03 | *LRP5L, CRYBB2P1* | 178  (117-258) | 3,570 | *LRP5L* is a member of the low-density lipoprotein receptor-related protein family, potentially influencing developmental processes, cell proliferation, and cholesterol homeostasis. *CRYBB2P1* is a pseudogene important for lens structure. |
| 20p12.1 | Deletion | 72 | 0.002 | 0.02 | *MACROD2* | 411  (256-621) | 8,220 | *MACROD2* encodes a mono-ADP-ribosylhydrolase involved in DNA repair and transcription regulation. *MACROD2* deletions are associated with neurodevelopmental disorders including autism and intellectual disability. |
| 7q31.1* | Deletion | 156 | 0.02 | 0.007 | *IMMP2L* | 167  (93-271)  202  (29-672) | 3,340  4,040 | *IMMP2L* encodes a mitochondrial inner membrane peptidase involved in mitochondrial protein processing and cellular energy metabolism*.* Variants are linked to neurodevelopmental disorders, including autism and ADHD,. |

**Table S2:** rCNVs deleting or duplicating disease-associated genes, including cytogenetic locations, frequencies, and evolutionary ages as estimated by the conservation of haplotypes flanking the rCNVs.

| 7p22.3 | Deletion | 72 | - | 0.03 | *SDK1* | 80  (55-111) | 1,598 | *SDK1* is a synaptic cell adhesion molecule involved in synapse formation and stability. It is associated with neurodevelopmental disorders including ASD and ADHD. |
| --- | --- | --- | --- | --- | --- | --- | --- | --- |
| 22q11.21 | Duplication | 354 | - | 0.03 | *USP18, DGCR6, PRODDH* | 113  (77-158) | 2,260 | *USP18* modulates interferon signaling and is linked to immune and neurological disorders. *DGCR6*, located in the DiGeorge syndrome critical region, influences neural crest cell migration and is implicated in DiGeorge syndrome. *PRODH* affects proline metabolism and is associated with hyperprolinemia and psychiatric conditions. |
| 19q13.4 | Deletion | 118 | - | 0.03 | *LILRA2, LILRA1, LILRB1, KIR3DX1* | 52  (38-70) | 1,046 | *LILRA1* and *LILRA2* are immune-related receptors affecting innate immunity, hematopoietic signaling, and neural plasticity. *LILRB1* is an inhibitory immune receptor that binds to MHC class I molecules and plays a key role in maintaining immune tolerance by suppressing excessive immune activation. *KIR3DX1* is a pseudogene involved in modulating natural killer (NK) cell responses via HLA class I recognition. |
| 1p36.21 | Deletion | 53 | - | 0.04 | *AADACL3, CFAP107, PRAMEF11* | 109  (76-149) | 2,180 | *AADACL3,* belongs to the serine esterase family and is involved in lipid metabolism. It has been implicated in metabolic disorders and cancer. *CFAP107*, is a structural component of cilia and flagella essential for flagellated sperm motility. *PRAMEF11 is* a member of the PRAME family, encoding leucine-rich repeat proteins expressed in the testis but also in various tumors. While their specific function is unclear, PRAME family members are known to act as transcriptional regulators involved in oncogenesis. |
| 2q37.3 | Deletion | 71 | 0.05 | - | *SCLY, ESPNL, KLHL30* | 448  (251-729) | 8,960 | *SCLY* is highly expressed in liver and kidney, maintaining selenium homeostasis and redox balance. Disruption of *SCLY* has been associated with metabolic conditions such as obesity, hyperinsulinemia, glucose intolerance, and hepatic steatosis. *ESPNL* encodes an actin-binding protein critical for the elongation and maintenance of stereocilia in auditory cells. *KLHL30* participates in protein ubiquitination via E3 ligase and is linked to Congenital Myopathy 23 and Nemaline Myopathy 8. Mutations in *KLHL* genes can disrupt muscle development and contribute to other pathological conditions due to their central role in protein regulation. |
| 6p21.33 | Deletion | 18 | 0.03 | - | *MUC22* | 1490  (1030-2070) | 29,800 | *MUC22* encodes a member of the mucin family glycoproteins that contribute to protective mucosal barriers. *MUC22* has been implicated in inflammatory responses and innate immunity. |
| 7p22.1 | Deletion | 159 | 0.08 | - | *FOXK1, AP5Z1, RADIL* | 879  (546-1330) | 17,580 | *FOXK1* is a transcription factor involved in the regulation of cell growth, muscle development, and metabolic pathways.  *AP5Z1* encodes a subunit of the adaptor protein complex AP5, essential for the sorting and trafficking of proteins within endosomes. Pathogenic variants of *AP5Z1* have been linked to hereditary spastic paraplegia. *RADIL* (Ras Association and DIL Domains) regulates integrin-mediated cell adhesion and migration by modulating the actin cytoskeleton. Disruptions of *RADIL* may lead to congenital disorders involving neural crest migration, various cancers due to altered cell motility, and neurodevelopmental disorders stemming from aberrant neuronal positioning. |
| 8q21.3 | Deletion | 66 | 0.04 | - | *LRRC69* | 666  (476-902) | 13,320 | *LRRC69* encodes a signal transduction protein involved in cell adhesion. *LRRC69* is primarily expressed in the testis and has been proposed as a risk factor for male infertility. |
| 13q33.2 | Deletion | 87 | 0.03 | - | *Non-genic region (near DAOA)* | 107  (70-156) | 2,140 | *DAOA* modulates D-amino acid oxidase activity thereby influencing NMDA receptor function. Through this mechanism, *DAOA* may contribute to altered glutamatergic signaling and increase susceptibility to multiple neuropsychiatric disorders. |
| 3q26.31 | Duplication | 42 | 0.04 | - | *NLGN1* | 148  (101-207) | 2,960 | *NLGN1* (neuroligin 1) is a member of the neuroligin family of neuronal cell surface proteins critical for synapse formation, maintenance, and function. *NLGN1* modulates excitatory synaptic efficacy and plasticity. Alterations in *NLGN1* have been implicated in autism spectrum disorder and various cognitive impairments. |

****** denotes different rCNVs at 7q31.1

| **Cytogenetic region** | **CNV type** | **Start (bp)** | **End (bp)** | **Size (Mb)** | **Carrier Frequency** | **Genes** |
| --- | --- | --- | --- | --- | --- | --- |
| 1p36.33 | Deletion | 776546 | 1114668 | 0.33 | 15 (0.04) | Multigenic |
| 1p36.33 | Deletion | 1138913 | 1179816 | 0.04 | 7 (0.02) | Multigenic |
| 1p36.33 | Deletion | 1550992 | 1564064 | 0.01 | 6 (0.01) | MIB2 |
| 1p36.31 | Deletion | 3381698 | 3417211 | 0.03 | 2 (0.005) | ARHGEF16, MEGF6 |
| 1p36.31 | Deletion | 6181605 | 6697530 | 0.51 | 9 (0.02) | Multigenic |
| 1p36.22 | Deletion | 12852872 | 12915847 | 0.06 | 4 (0.01) | PRAMEF1, RAMEF11, HNRNPCL1 |
| 1p36.21 | Deletion | 16015784 | 16188681 | 0.17 | 16 (0.04) | Multigenic |
| 1p36.13 | Deletion | 17249219 | 17320122 | 0.07 | 2 (0.005) | CROCC, MFAP2 |
| 1p35.2 | Deletion | 30979276 | 31012257 | 0.03 | 3 (0.008) | Non-genic region |
| 1p31.1 | Deletion | 55515618 | 55532142 | 0.01 | 2 (0.005) | PCSK9, USP24 |
| 1p31.1 | Deletion | 74492543 | 74649321 | 0.15 | 4 (0.01) | LRRIQ3 |
| 1p22.2 | Duplication | 89265248 | 89302955 | 0.03 | 8 (0.02) | PKN2 |
| 1p22.2 | Deletion | 91739270 | 91916880 | 0.17 | 3 (0.008) | HFM1 |
| 1p22.1 | Deletion | 93651547 | 93724389 | 0.07 | 3 (0.008) | CCDC18 |
| 1p21.1 | Duplication | 103354135 | 103548505 | 0.19 | 3 (0.008) | COL11A1 |
| 1p13.3* | Deletion | 109789795 | 109820919 | 0.03 | 5 (0.014) | CELSR2 |
| 1q21.1 | Duplication | 145610210 | 145762959 | 0.15 | 6 (0.01) | Multigenic |
| 1q21.3 | Deletion | 151239051 | 151263530 | 0.02 | 3 (0.008) | PSMD4, ZNF687 |
| 1q21.3 | Deletion | 152526812 | 152552461 | 0.02 | 7 (0.02) | LCE3E, LCE3D |
| 1q22 | Deletion | 155151048 | 155165892 | 0.01 | 2 (0.005) | TRIM46, MUC1, THBS3 |
| 1q25.3-q31.1 | Duplication | 185565704 | 186151390 | 0.58 | 7 (0.02) | HMCN1 |
| 1q31.3 | Deletion | 194083813 | 194564447 | 0.48 | 3 (0.008) | Non-genic region |
| 1q31.3 | Duplication | 197019858 | 197644773 | 0.62 | 10 (0.02) | Multigenic |
| 1q31.3 | Duplication | 197892733 | 198544730 | 0.65 | 2 (0.005) | LHX9, NEK7, ATP6V1G3 |
| 1q32.1 | Duplication | 201415592 | 201603081 | 0.18 | 7 (0.02) | PHLDA3, CSRP1 |
| 1q42.12 | Deletion | 225061843 | 225539363 | 0.47 | 2 (0.005) | DNAH14 |
| 1q42.13 | Deletion | 228520973 | 228564884 | 0.04 | 14 (0.04) | OBSCN |
| 1q42.13 | Duplication | 230372099 | 230429143 | 0.05 | 7 (0.02) | GALNT2 |
| 1q44 | Deletion | 245848798 | 245861522 | 0.01 | 7 (0.02) | KIF26B |
| 2p23.3 | Deletion | 27292534 | 27360863 | 0.06 | 9 (0.02) | Multigenic |
| 2p22.2 | Duplication | 38510608 | 38580233 | 0.07 | 13 (0.03) | ATL2 |
| 2p16.3 | Deletion | 49676328 | 49764298 | 0.08 | 9 (0.02) | Non-genic region |

**Table S3:** Cytogenetic region, CNV type, genomic coordinates (base pairs), CNV size (megabases), carrier frequency, and genes affected by rCNVs detected in Southwest Indians.

| 2p16.1 | Duplication | 57399830 | 57445335 | 0.04 | 3 (0.008) | Non-genic region |
| --- | --- | --- | --- | --- | --- | --- |
| 2p15 | Deletion | 61454010 | 61566536 | 0.11 | 2 (0.005) | USP34 |
| 2p13.1 | Deletion | 73847024 | 73900329 | 0.05 | 3 (0.008) | NAT8, ALMS1P |
| 2q12.3 | Duplication | 107830992 | 107862981 | 0.03 | 2 (0.005) | Non-genic region |
| 2q12.3 | Deletion | 109085455 | 109098854 | 0.01 | 3 (0.008) | GCC2 |
| 2q13 | Duplication | 110852875 | 110980346 | 0.12 | 8 (0.02) | MALL, NPHP1 |
| 2q14.3 | Deletion | 127804699 | 127834459 | 0.02 | 2 (0.005) | BIN1 |
| 2q14.3 | Deletion | 128327387 | 128471218 | 0.14 | 2 (0.005) | Multigenic |
| 2q21.1* | Deletion | 132057166 | 132298468 | 0.24 | 5 (0.014) | Multigenic |
| 2q31.2 | Duplication | 179365400 | 179618462 | 0.25 | 2 (0.005) | PLEKHA3, TTN |
| 2q33.1 | Duplication | 201397345 | 201450068 | 0.05 | 11 (0.03) | SGOL2 |
| 2q37.3 | Deletion | 238999875 | 239071623 | 0.07 | 35 (0.10) | SCLY, ESPNL, KLHL30 |
| 2q37.3 | Duplication | 241800707 | 242212672 | 0.41 | 2 (0.005) | Multigenic |
| 3p26.3 | Duplication | 61495 | 382547 | 0.32 | 9 (0.02) | CHL1 |
| 3p25.2* | Duplication | 12628920 | 12806123 | 0.17 | 19 (0.05) | RAF1, TMEM40 |
| 3p24.3 | Duplication | 21083986 | 21144408 | 0.06 | 5 (0.01) | Non-genic region |
| 3q12.2 | Duplication | 100351696 | 100441716 | 0.09 | 2 (0.005) | GPR128, TFG |
| 3q13.13 | Deletion | 108254751 | 108301920 | 0.04 | 5 (0.01) | KIAA1524 |
| 3q25.32 | Deletion | 158515499 | 158539294 | 0.02 | 2 (0.005) | MFSD1 |
| 3q26.1 | Deletion | 164709997 | 164750447 | 0.04 | 3 (0.008) | SI |
| 3q26.31 | Duplication | 172321698 | 172397868 | 0.07 | 3 (0.008) | NCEH1 |
| 3q26.31 | Duplication | 173239453 | 173281958 | 0.04 | 28 (0.08) | NLGN1 |
| 3q26.33 | Deletion | 180320704 | 180386213 | 0.06 | 3 (0.008) | TTC14, CCDC39 |
| 4p16.3 | Deletion | 942006 | 1064557 | 0.12 | 29 (0.08) | Multigenic |
| 4p16.1 | Deletion | 8212524 | 8239393 | 0.02 | 18 (0.05) | SH3TC1 |
| 4p12 | Duplication | 46038274 | 46086060 | 0.04 | 3 (0.008) | GABRG1 |
| 4q12* | Duplication | 58604281 | 58770663 | 0.16 | 7 (0.02) | Non-genic region |
| 4q22.1 | Deletion | 92930924 | 93088664 | 0.15 | 12 (0.03) | Non-genic region |
| 4q35.1 | Deletion | 186322002 | 186381059 | 0.05 | 2 (0.005) | UFSP2, CCDC110 |
| 4q35.2 | Duplication | 189139613 | 189388320 | 0.24 | 3 (0.008) | Non-genic region |
| 4q35.2 | Deletion | 189519649 | 189557490 | 0.03 | 2 (0.005) | Non-genic region |
| 5p15.1 | Deletion | 17697085 | 17745762 | 0.04 | 13 (0.03) | Non-genic region |
| 5p13.3 | Duplication | 32101400 | 32159517 | 0.05 | 19 (0.05) | PDZD2, GOLPH3 |
| 5p12-p11 | Duplication | 45602976 | 46399093 | 0.79 | 7 (0.02) | HCN1 |
| 5q31.1 | Duplication | 130736800 | 130840385 | 0.10 | 11 (0.03) | RAPGEF6 |
| 5q31.1 | Deletion | 132150484 | 132161784 | 0.01 | 11 (0.03) | SOWAHA, SHROOM1 |
| 5q31.3 | Deletion | 140165568 | 140264271 | 0.09 | 20 (0.05) | PCDHA1-13 |
| 5q32 | Duplication | 146687393 | 146817347 | 0.13 | 3 (0.008) | STK32A, DPYSL3 |
| 5q35.3 | Deletion | 176730190 | 176958397 | 0.22 | 14 (0.04) | Multigenic |
| 5q35.3 | Deletion | 179200919 | 179308012 | 0.11 | 7 (0.02) | Multigenic |
| 6p22.3 | Deletion | 16186284 | 16326842 | 0.14 | 6 (0.01) | GMPR, ATXN1 |
| 6p22.2 | Deletion | 26020276 | 26056321 | 0.03 | 5 (0.01) | Multigenic |
| 6p22.1 | Duplication | 29094696 | 29161435 | 0.06 | 4 (0.01) | OR2J2 |
| 6p22.1 | Deletion | 29849619 | 29896421 | 0.04 | 7 (0.02) | HLA-H, HLA-G, HLA-J |
| 6p21.33 | Deletion | 30969527 | 30987786 | 0.01 | 25 (0.07) | MUC22 |
| 6p21.33* | Deletion | 31355318 | 31453640 | 0.09 | 93 (0.26) | MICA, HCP5, PMSP, HCG26 |
| 6p21.31 | Deletion | 34480637 | 34544384 | 0.06 | 5 (0.01) | PACSIN1, SPDEF |
| 6q11.1-q12 | Duplication | 63136576 | 63610969 | 0.47 | 10 (0.02) | Non-genic region |
| 6q22.31 | Duplication | 124681503 | 124900701 | 0.21 | 9 (0.02) | NKAIN2 |
| 7p22.3 | Duplication | 44935 | 68920 | 0.02 | 15 (0.04) | Non-genic region |
| 7p22.3 | Duplication | 44935 | 81237 | 0.03 | 6 (0.01) | Non-genic region |
| 7p22.3 | Duplication | 44935 | 162145 | 0.11 | 7 (0.02) | Non-genic region |
| 7p22.3 | Duplication | 44935 | 171613 | 0.12 | 8 (0.02) | Non-genic region |
| 7p22.1 | Deletion | 4721171 | 4880954 | 0.15 | 56 (0.16) | FOXK1, AP5Z1, RADIL |
| 7p22.3 | Deletion | 9680160 | 9858697 | 0.17 | 7 (0.02) | Non-genic region |
| 7p12.3 | Duplication | 47496258 | 47619815 | 0.12 | 3 (0.008) | TNS3 |
| 7q11.23* | Duplication | 76131646 | 76639871 | 0.51 | 6 (0.01) | Multigenic |
| 7q22.1 | Duplication | 100634386 | 100686356 | 0.05 | 7 (0.02) | MUC12, MUC17 |
| 7q31.1* | Deletion | 110971919 | 111309224 | 0.33 | 16 (0.04) | IMMP2L |
| 7q31.1 | Duplication | 113207209 | 113493772 | 0.28 | 2 (0.005) | Non-genic region |
| 7p36.1 | Deletion | 149410638 | 149430847 | 0.02 | 26 (0.07) | KRBA1 |
| 7p36.1 | Deletion | 150639871 | 150883937 | 0.24 | 6 (0.01) | Multigenic |
| 8p23.3 | Duplication | 1697573 | 1753497 | 0.05 | 13 (0.03) | CLN8 |
| 8p21.3 | Deletion | 21959728 | 21997479 | 0.03 | 11 (0.03) | FAM160B2, NUDT18, HR, REEP4 |
| 8p12 | Duplication | 30699708 | 30731171 | 0.03 | 8 (0.02) | TEX15 |
| 8q21.3 | Deletion | 92114936 | 92181214 | 0.06 | 31 (0.08) | LRRC69 |
| 8q22.2-q22.3 | Duplication | 101421798 | 101612659 | 0.19 | 6 (0.01) | ANKRD46, SNX31 |
| 8q24.3 | Deletion | 145101381 | 145114919 | 0.01 | 2 (0.005) | SPATC1, OPLAH |
| 9p24.3* | Duplication | 483018 | 489338 | 0.006 | 2 (0.005) | KANK1 |
| 9p24.2 | Duplication | 3138608 | 3267584 | 0.12 | 3 (0.008) | RFX3 |
| 9p24.1 | Duplication | 6587200 | 6928789 | 0.34 | 22 (0.06) | GLDC, KDM4C |
| 9p23 | Deletion | 12022175 | 12165758 | 0.14 | 6 (0.01) | Non-genic region |
| 9q34.12 | Deletion | 133759566 | 133816023 | 0.05 | 5 (0.01) | ABL1, QRFP, FIBCD1 |
| 10p14 | Deletion | 11527215 | 11582344 | 0.05 | 4 (0.01) | USP6NL |
| 10p11.21 | Duplication | 37487831 | 37529216 | 0.04 | 2 (0.005) | ANKRD30A |
| 10q11.22 | Duplication | 47412588 | 47650490 | 0.23 | 6 (0.01) | FAM35DP |
| 10q21.1 | Deletion | 57138742 | 57650954 | 0.51 | 2 (0.005) | PCDH15, MTRNR2L5 |
| 10q23.33 | Deletion | 94814907 | 94834581 | 0.01 | 6 (0.01) | EXOC6, CYP26C1, CYP26A1 |
| 10q24.1 | Duplication | 98792520 | 98876941 | 0.08 | 2 (0.005) | SLIT1 |
| 10q25.3 | Deletion | 116930870 | 117575619 | 0.64 | 2 (0.005) | ATRNL1 |
| 11p15.5 | Deletion | 280464 | 1015021 | 0.73 | 4 (0.01) | Multigenic |
| 11p15.5 | Deletion | 2386636 | 2469915 | 0.08 | 9 (0.02) | CD81, TSSC4, TRPM5, KCNQ1 |
| 11q22.3 | Deletion | 102937178 | 103191959 | 0.25 | 3 (0.008) | DCUN1D5, DYNC2H1 |
| 11q22.3 | Deletion | 108097333 | 108352891 | 0.25 | 2 (0.005) | ATM, KDELC2 |
| 11q23.2 | Deletion | 113267957 | 113280724 | 0.01 | 7 (0.02) | ANKK1, DRD2 |
| 11q25 | Duplication | 134792477 | 134934063 | 0.14 | 4 (0.01) | Non-genic region |
| 12p13.31* | Duplication | 8000912 | 8114429 | 0.11 | 2 (0.005) | SLC2A14, NANOGP1, SLC2A3 |
| 12p13.31 | Duplication | 9435244 | 9664352 | 0.22 | 16 (0.04) | DDX12P |
| 12p11.11 | Deletion | 53439537 | 53454938 | 0.01 | 9 (0.02) | TENC1 |
| 12q14.2* | Duplication | 63946056 | 64118558 | 0.17 | 2 (0.005) | DPY19L2 |
| 12q21.31 | Duplication | 85070164 | 85755820 | 0.68 | 7 (0.02) | SLC6A15, TSPAN19, LRRIQ1, ALX1 |
| 13q12.11 | Duplication | 21728134 | 21746637 | 0.01 | 22 (0.06) | SKA3 |
| 13q14.11 | Duplication | 43718991 | 43828170 | 0.10 | 9 (0.02) | ENOX1 |
| 13q22.1 | Deletion | 73331944 | 73575455 | 0.24 | 2 (0.005) | DIS3, PIBF1 |
| 13q31.1* | Deletion | 84101480 | 84157927 | 0.05 | 51 (0.14) | Non-genic region |
| 13q33.1 | Duplication | 103382660 | 103402638 | 0.02 | 9 (0.02) | CCDC168 |
| 13q33.2* | Deletion | 106219874 | 106307349 | 0.08 | 25 (0.07) | Non-genic region |
| 14q11.2 | Duplication | 19700048 | 20416499 | 0.71 | 11 (0.03) | Multigenic |
| 14q11.2 | Duplication | 20213937 | 20416499 | 0.20 | 2 (0.005) | Multigenic |
| 14q11.2 | Deletion | 21358155 | 21393289 | 0.03 | 18 (0.05) | RNASE3, ECRP, RNASE2 |
| 14q12 | Deletion | 27596242 | 27714462 | 0.11 | 6 (0.01) | Non-genic region |
| 14q22.1 | Duplication | 52354228 | 52416412 | 0.06 | 4 (0.01) | GNG2 |
| 14q23.1 | Deletion | 58666970 | 59006831 | 0.33 | 3 (0.008) | Multigenic |
| 14q23.1 | Duplication | 60903572 | 60938407 | 0.03 | 10 (0.02) | Non-genic region |
| 14q31.3 | Duplication | 89171711 | 89466033 | 0.29 | 4 (0.01) | EML5, TTC8 |
| 14q32.33 | Deletion | 104546893 | 104662014 | 0.11 | 3 (0.008) | ASPG, KIF26A |
| 15q11.2 | Deletion | 22750305 | 23272733 | 0.52 | 3 (0.008) | Multigenic |
| 15q11.2 | Duplication | 24357212 | 24472002 | 0.11 | 6 (0.01) | PWRN2 |
| 15q15.1 | Deletion | 42104826 | 42185482 | 0.08 | 18 (0.05) | Multigenic |
| 15q15.1 | Deletion | 42143533 | 42164078 | 0.02 | 6 (0.01) | SPTBN5 |
| 15q15.2 | Deletion | 42869028 | 42934631 | 0.06 | 6 (0.01) | STARD9 |
| 15q21.3 | Deletion | 57720473 | 57754873 | 0.03 | 2 (0.005) | CGNL1 |
| 15q22.2 | Deletion | 62128861 | 62332980 | 0.20 | 10 (0.02) | VPS13C |
| 15q26.3 | Duplication | 102264489 | 102461162 | 0.19 | 11 (0.03) | Multigenic |
| 16p13.3 | Deletion | 514407 | 875050 | 0.36 | 32 (0.09) | Multigenic |
| 16p13.3 | Deletion | 678430 | 726120 | 0.04 | 7 (0.02) | Multigenic |
| 16p13.3 | Deletion | 767179 | 780615 | 0.01 | 3 (0.008) | Multigenic |
| 16p13.3 | Deletion | 812700 | 825628 | 0.01 | 4 (0.01) | MSLN |
| 16p13.3 | Deletion | 1799407 | 1858888 | 0.05 | 19 (0.05) | Multigenic |
| 16p13.3 | Deletion | 2003399 | 2087547 | 0.08 | 22 (0.06) | Multigenic |
| 16p13.3 | Deletion | 2130189 | 2199788 | 0.06 | 3 (0.008) | Multigenic |
| 16p13.3 | Deletion | 2510603 | 2788281 | 0.27 | 5 (0.01) | Multigenic |
| 16p13.3 | Deletion | 3170210 | 3191356 | 0.02 | 3 (0.008) | ZNF205, ZNF213 |
| 16p13.2 | Deletion | 8531584 | 8615618 | 0.08 | 4 (0.01) | TMEM114 |
| 16q22.1 | Deletion | 67180198 | 67320972 | 0.14 | 9 (0.02) | Multigenic |
| 16q24.1 | Deletion | 84209816 | 84233596 | 0.02 | 4 (0.01) | DNAAF1, TAF1C, ADAD2 |
| 16q24.2 | Deletion | 88440878 | 88601281 | 0.16 | 5 (0.01) | ZNF469, ZFPM1 |
| 17p11.2 | Duplication | 21319849 | 21503147 | 0.18 | 3 (0.008) | KCNJ12 |
| 17p11.2-p11.1* | Deletion | 21539613 | 22242355 | 0.70 | 18 (0.05) | UBBP4, FAM27L, MTRNR2L1 |
| 17q12 | Duplication | 34443811 | 34466631 | 0.02 | 22 (0.06) | Non-genic region |
| 17q25.3 | Duplication | 80391684 | 80544855 | 0.15 | 2 (0.005) | Multigenic |
| 18q21.1 | Deletion | 63203008 | 63275241 | 0.07 | 7 (0.02) | Non-genic region |
| 19q13.33 | Duplication | 50520133 | 50548187 | 0.02 | 10 (0.02) | VRK3, ZNF473 |
| 19q13.41 | Duplication | 53518590 | 53548950 | 0.03 | 22 (0.06) | ERVV-1, ERVV-2 |
| 19q13.42* | Deletion | 53932295 | 54011384 | 0.07 | 3 (0.008) | TPM3P9, ZNF761, ZNF813 |
| 20p12.1* | Deletion | 14815778 | 15171838 | 0.35 | 2 (0.005) | MACROD2 |
| 20q11.22 | Deletion | 33566722 | 33591284 | 0.02 | 10 (0.02) | MYH7B, TRPC4AP |
| 20q13.33 | Deletion | 58416428 | 58500453 | 0.08 | 11 (0.03) | PHACTR3, SYCP2 |
| 20q13.33 | Deletion | 60788970 | 60912731 | 0.12 | 10 (0.02) | Multigenic |
| 20q13.33 | Deletion | 62028210 | 62836184 | 0.80 | 7 (0.02) | Multigenic |
| 20q13.33 | Deletion | 62117518 | 62203708 | 0.08 | 7 (0.02) | Multigenic |
| 20q13.33 | Deletion | 62187669 | 62202150 | 0.01 | 4 (0.01) | HELZ2 |
| 20q13.33 | Deletion | 62193253 | 62722711 | 0.52 | 3 (0.008) | Multigenic |
| 22q11.22 | Deletion | 22381327 | 22538738 | 0.15 | 3 (0.008) | Non-genic region |
| 22q11.22 | Deletion | 22394144 | 23237674 | 0.84 | 8 (0.02) | Multigenic |
| 22q11.22 | Duplication | 22508432 | 22734671 | 0.22 | 2 (0.005) | Multigenic |
| 22q11.22 | Deletion | 22599238 | 22946825 | 0.34 | 6 (0.01) | Multigenic |
| 22q11.22 | Deletion | 22770917 | 23010666 | 0.23 | 7 (0.02) | Multigenic |
| 22q11.23-q12.1* | Duplication | 25650406 | 25910667 | 0.26 | 79 (0.22) | IGLL3P, LRP5L, CRYBB2P1 |

*Found in PI

**Table S4:** Cytogenetic region, CNV type, genomic coordinates (base pairs), CNV size (megabases), carrier frequency, and genes affected by rCNVs detected in Plains Indians.

| **Cytogenetic region** | **CNV type** | **Start (bp)** | **End (bp)** | **Size (Mb)** | **Carrier Frequency** | **Genes** |
| --- | --- | --- | --- | --- | --- | --- |
| 1p36.32 | Duplication | 3211458 | 3249242 | 0.03 | 8 (0.02) | PRDM16 |
| 1p36.21 | Deletion | 12783336 | 12836483 | 0.05 | 31 (0.08) | AADACL3, CFAP107, PRAMEF12 |
| 1p36.13 | Duplication | 18027377 | 18650794 | 0.62 | 8 (0.02) | ACTL8, IGSF21 |
| 1p22.2 | Deletion | 90304817 | 90313153 | 0.008 | 7 (0.01) | LRRC8D |
| 1p13.3 | Deletion | 109201183 | 109505884 | 0.30 | 3 (0.007) | Multigenic |
| 1p13.3* | Deletion | 109705022 | 109832283 | 0.12 | 2 (0.005) | CELSR2 |
| 1q21.3 | Deletion | 150797134 | 150819989 | 0.02 | 95 (0.24) | ARNT |
| 1q31.1 | Duplication | 187261219 | 187478245 | 0.21 | 4 (0.01) | Non-genic region |
| 1q31.3 | Duplication | 194977690 | 195163711 | 0.18 | 3 (0.007) | Non-genic region |
| 1q43 | Duplication | 239896209 | 240067411 | 0.17 | 3 (0.007) | Multigenic |
| 2p16.3 | Deletion | 52035257 | 52074452 | 0.03 | 9 (0.02) | Non-genic region |
| 2p12 | Deletion | 89390395 | 89877778 | 0.48 | 21 (0.05) | IGKV2-28, IGKV4-1 |
| 2q14.3 | Deletion | 128050883 | 128122391 | 0.07 | 3 (0.007) | ERCC3 |
| 2q21.1* | Deletion | 132077379 | 132311088 | 0.23 | 1 (0.002) | Multigenic |
| 2q24.2 | Duplication | 160317408 | 160451108 | 0.13 | 4 (0.01) | BAZ2B |
| 2q24.3 | Duplication | 169205375 | 169238400 | 0.03 | 8 (0.02) | Non-genic region |
| 3p26.1 | Duplication | 5899967 | 6115317 | 0.21 | 3 (0.007) | Non-genic region |
| 3p25.2* | Duplication | 12610706 | 12792622 | 0.18 | 43 (0.11) | MKRN2, RAF1, TMEM40 |
| 3p24.3 | Duplication | 20167830 | 20951147 | 0.78 | 5 (0.01) | KAT2B, SGOL1 |
| 3p14.1 | Deletion | 65159939 | 65187636 | 0.02 | 24 (0.06) | Non-genic region |
| 3p12.1 | Deletion | 84832445 | 84882133 | 0.05 | 7 (0.01) | Non-genic region |
| 3q29 | Duplication | 193668899 | 194233500 | 0.56 | 4 (0.01) | Multigenic |
| 4p15.2 | Deletion | 22155404 | 22244178 | 0.08 | 5 (0.01) | Non-genic region |
| 4q12* | Duplication | 58604281 | 58758826 | 0.15 | 1 (0.002) | Non-genic region |
| 4q28.1 | Deletion | 127597069 | 127667944 | 0.07 | 9 (0.02) | Non-genic region |
| 4q28.3 | Deletion | 136751948 | 136819237 | 0.06 | 7 (0.01) | Non-genic region |
| 5p15.33 | Duplication | 1052360 | 1275528 | 0.22 | 9 (0.02) | SLC12A7, SLC6A19, SLC6A18, TERT |
| 5p15.31 | Deletion | 8753487 | 8796390 | 0.04 | 8 (0.02) | Non-genic region |
| 5p15.2 | Duplication | 10785424 | 11313232 | 0.52 | 3 (0.007) | CTNND2 |
| 5p12 | Duplication | 45905997 | 46348483 | 0.44 | 14 (0.03) | Non-genic region |
| 5q13.3 | Duplication | 73999205 | 74066386 | 0.06 | 18 (0.04) | HEXB, GFM2, NSA2 |
| 5q23.1 | Deletion | 119867182 | 120009805 | 0.14 | 3 (0.007) | PRR16 |
| 5q35.3 | Duplication | 178728830 | 178935665 | 0.20 | 2 (0.005) | ADAMTS2 |

| 6p24.3 | Duplication | 7187246 | 7355518 | 0.16 | 2 (0.005) | RREB1, SSR1, CAGE1 |
| --- | --- | --- | --- | --- | --- | --- |
| 6p21.33* | Deletion | 31355318 | 31451476 | 0.09 | 60 (0.15) | MICA, HCG-26, HCP-5, PMSP |
| 6q12 | Deletion | 65577402 | 65837939 | 0.26 | 3 (0.007) | EYS |
| 6q12 | Deletion | 67075448 | 67105019 | 0.03 | 9 (0.02) | Non-genic region |
| 6q14.1 | Deletion | 74620278 | 74719628 | 0.09 | 2 (0.005) | Non-genic region |
| 6q14.1 | Deletion | 78973201 | 79031742 | 0.05 | 26 (0.06) | Non-genic region |
| 6q14.1 | Deletion | 142156294 | 142236578 | 0.08 | 11 (0.02) | Non-genic region |
| 6q26 | Deletion | 162765089 | 162809009 | 0.04 | 7 (0.01) | PRKN |
| 7p22.3 | Duplication | 140736 | 295765 | 0.15 | 2 (0.005) | Non-genic region |
| 7p22.2 | Deletion | 3545955 | 3618143 | 0.07 | 28 (0.07) | SDK1 |
| 7p21.2 | Duplication | 13841798 | 13972307 | 0.13 | 2 (0.005) | ETV1 |
| 7p21.2 | Deletion | 14113503 | 14231680 | 0.11 | 10 (0.02) | DGKB |
| 7p15.3 | Duplication | 21523467 | 21651293 | 0.12 | 13 (0.03) | SP4, DNAH11 |
| 7p12.1 | Deletion | 53460686 | 53590250 | 0.13 | 9 (0.02) | Non-genic region |
| 7p11.2 | Deletion | 54938083 | 55072814 | 0.13 | 4 (0.01) | Non-genic region |
| 7q11.1-q11.21 | Duplication | 61075979 | 62060344 | 0.98 | 9 (0.02) | Non-genic region |
| 7q11.21 | Deletion | 64679561 | 64956181 | 0.27 | 3 (0.007) | ZNF117, INTS4P1, ZNF92 |
| 7q11.23* | Duplication | 76066189 | 76557212 | 0.49 | 1 (0.002) | Multigenic |
| 7q31.1* | Deletion | 111085618 | 111242161 | 0.15 | 6 (0.01) | IMMP2L |
| 7q34 | Deletion | 141407907 | 141441259 | 0.03 | 3 (0.007) | WEE2, SSBP1 |
| 7q36.2 | Deletion | 154062113 | 154088591 | 0.02 | 14 (0.03) | DPP6 |
| 7q36.3 | Duplication | 157707876 | 157860945 | 0.15 | 7 (0.01) | PTPRN2 |
| 8p23.2 | Deletion | 4692648 | 4711611 | 0.01 | 2 (0.005) | CSMD1 |
| 8p23.2 | Deletion | 5713881 | 5803581 | 0.09 | 25 (0.06) | Non-genic region |
| 8p23.2 | Deletion | 6017976 | 6032282 | 0.01 | 6 (0.01) | Non-genic region |
| 8p23.1 | Duplication | 6490665 | 6554615 | 0.06 | 3 (0.007) | MCPH1 |
| 8p23.1 | Duplication | 6867213 | 6994825 | 0.12 | 3 (0.007) | DEFA3, DEFA5 |
| 8p22 | Deletion | 13643825 | 13653134 | 0.009 | 4 (0.01) | Non-genic region |
| 8p11.1 | Duplication | 43708027 | 43910848 | 0.20 | 3 (0.007) | Non-genic region |
| 8p11.1 | Duplication | 47224322 | 47309786 | 0.08 | 2 (0.005) | Non-genic region |
| 8p11.1 | Duplication | 47338235 | 47947701 | 0.60 | 2 (0.005) | Non-genic region |
| 8p11.1 | Duplication | 47463201 | 47621326 | 0.15 | 6 (0.01) | Non-genic region |
| 8p11.1 | Duplication | 47582366 | 48066866 | 0.48 | 6 (0.01) | Non-genic region |
| 8q13.3 | Duplication | 71707472 | 71800113 | 0.09 | 2 (0.005) | Non-genic region |
| 8q22.3 | Deletion | 103907253 | 104078514 | 0.17 | 3 (0.007) | ATP6V1C1 |
| 8q24.23 | Deletion | 137687955 | 137851655 | 0.16 | 6 (0.01) | Non-genic region |
| 9p24.3 | Deletion | 36587 | 219826 | 0.18 | 2 (0.005) | Multigenic |
| 9p24.3 | Duplication | 246995 | 360439 | 0.11 | 4 (0.01) | DOCK8 |
| 9p24.3* | Duplication | 526772 | 704075 | 0.17 | 7 (0.01) | KANK1 |
| 9p23 | Deletion | 11883047 | 11950277 | 0.06 | 2 (0.005) | Non-genic region |
| 9p23 | Deletion | 11903938 | 12179991 | 0.27 | 27 (0.06) | Non-genic region |
| 9p22.3 | Deletion | 14578049 | 14666870 | 0.08 | 2 (0.005) | ZDHHC21 |
| 9p21.31 | Duplication | 83636366 | 83674448 | 0.03 | 2 (0.005) | Non-genic region |
| 9q34.11 | Deletion | 131689678 | 131843027 | 0.15 | 2 (0.005) | Multigenic |
| 10p14 | Deletion | 6661779 | 6717773 | 0.05 | 7 (0.01) | Non-genic region |
| 10q11.22 | Deletion | 46410734 | 47173619 | 0.76 | 2 (0.005) | Multigenic |
| 10q11.22 | Deletion | 81577614 | 81972386 | 0.39 | 5 (0.01) | Multigenic |
| 10q24.2 | Duplication | 100278017 | 100344136 | 0.06 | 3 (0.007) | HPSE2 |
| 10q26.12 | Deletion | 122759878 | 122774992 | 0.01 | 2 (0.005) | Non-genic region |
| 10q26.3 | Duplication | 135266389 | 135377448 | 0.11 | 2 (0.005) | SCART1, CYP2E1, SYCE1 |
| 11p14.3 | Deletion | 24042316 | 24094782 | 0.05 | 10 (0.02) | Non-genic region |
| 11p14.3 | Deletion | 24085740 | 24138206 | 0.05 | 18 (0.04) | Non-genic region |
| 8p23.2 | Deletion | 6017976 | 6032282 | 0.01 | 6 (0.01) | Non-genic region |
| 11p14.3 | Deletion | 25618242 | 25755821 | 0.13 | 2 (0.005) | Non-genic region |
| 11p12 | Duplication | 50343409 | 51228612 | 0.88 | 8 (0.02) | LOC646813 |
| 11q11 | Deletion | 55127597 | 55204003 | 0.07 | 86 (0.22) | OR4A15 |
| 11q21 | Duplication | 95970204 | 96229007 | 0.25 | 3 (0.007) | MAML2, CCDC82 |
| 11q25 | Duplication | 133677241 | 133715739 | 0.03 | 6 (0.01) | Non-genic region |
| 11q25 | Duplication | 134234452 | 134583198 | 0.34 | 3 (0.007) | Multigenic |
| 12p13.31* | Duplication | 7996890 | 8123306 | 0.12 | 4 (0.01) | NANOG, SLC2A14, NECAP1, CLEC4A |
| 12p12.1 | Deletion | 21270899 | 21319219 | 0.04 | 8 (0.02) | SLCO1B1 |
| 12p11.1 | Duplication | 33862326 | 34819926 | 0.95 | 4 (0.01) | ALG10 |
| 12q14.2* | Duplication | 63947694 | 64129108 | 0.18 | 2 (0.005) | DPY19L2 |
| 12q14.3 | Deletion | 67122427 | 67305277 | 0.18 | 4 (0.01) | GRIP1 |
| 12q24.22 | Deletion | 118010309 | 118032478 | 0.02 | 2 (0.005) | KSR2 |
| 12q24.33 | Deletion | 131730317 | 131814934 | 0.08 | 5 (0.01) | Non-genic region |
| 13q13.3 | Deletion | 35597170 | 36202269 | 0.60 | 2 (0.005) | NBEA |
| 13q31.1* | Deletion | 84102440 | 84157927 | 0.05 | 80 (0.20) | Non-genic region |
| 13q31.1-q31.2 | Duplication | 85726019 | 88158837 | 2.43 | 10 (0.02) | SLITRK6 |
| 13q31.3 | Deletion | 94449198 | 94809726 | 0.36 | 2 (0.005) | GPC6 |
| 13q33.2* | Deletion | 105918084 | 106007135 | 0.08 | 2 (0.005) | Non-genic region |
| 14q21.1 | Duplication | 42717951 | 43127730 | 0.41 | 7 (0.01) | Non-genic region |
| 14q22.2 | Duplication | 53983732 | 54528067 | 0.54 | 3 (0.007) | BMP4 |
| 14q32.33 | Deletion | 106926025 | 107103272 | 0.17 | 16 (0.04) | IGHM |
| 15q11.1-q11.2 | Duplication | 20306549 | 20777695 | 0.47 | 6 (0.01) | Multigenic |
| 15q13.3 | Duplication | 32020066 | 32514926 | 0.49 | 13 (0.03) | OTUD7A, CHRNA7 |
| 15q22.2 | Duplication | 59942574 | 60127418 | 0.18 | 3 (0.007) | BNIP2 |
| 15q22.2 | Duplication | 62338797 | 62414014 | 0.07 | 3 (0.007) | VPS13C, C2CD4A |
| 17p11.2-p11.1* | Duplication | 21704418 | 22242355 | 0.53 | 19 (0.04) | Multigenic |
| 17q25.3 | Duplication | 77365079 | 77393602 | 0.02 | 4 (0.01) | RBFOX3 |
| 11p14.3 | Deletion | 25618242 | 25755821 | 0.13 | 2 (0.005) | Non-genic region |
| 18p11.32 | Deletion | 1913316 | 1980668 | 0.06 | 3 (0.007) | Non-genic region |
| 18q22.1 | Deletion | 66746208 | 66755508 | 0.009 | 8 (0.02) | Non-genic region |
| 19q13.2-q13.31 | Duplication | 43322065 | 43539189 | 0.21 | 9 (0.02) | PSG3, PSG1, PSG6, PSG7, PSG11 |
| 19q13.42* | Deletion | 53932295 | 54014178 | 0.08 | 2 (0.005) | ZNF761, ZNF813 |
| 19q13.42 | Deletion | 55030183 | 55148487 | 0.11 | 28 (0.07) | KIR3DX1, LILRA2, LILRA1, LILRB1 |
| 20p12.1* | Deletion | 14758111 | 14830453 | 0.07 | 18 (0.04) | MACROD2 |
| 21q21.1 | Deletion | 20509659 | 20712858 | 0.20 | 8 (0.02) | Non-genic region |
| 21q21.3 | Deletion | 31292936 | 31382068 | 0.08 | 4 (0.01) | GRIK1 |
| 22q11.21 | Duplication | 18642145 | 18996565 | 0.35 | 30 (0.07) | USP18, DGCR6, PRODDH |
| 22q11.22 | Deletion | 22386617 | 23257051 | 0.87 | 7 (0.01) | Multigenic |
| 22q11.22 | Deletion | 22512415 | 23224058 | 0.71 | 7 (0.01) | Multigenic |
| 22q11.22 | Deletion | 22681312 | 23224058 | 0.54 | 26 (0.06) | Multigenic |
| 22q11.22 | Deletion | 22740855 | 23224058 | 0.48 | 21 (0.05) | ZNF280B, ZNF280A, PRAME, GGTLC2 |
| 22q11.22 | Deletion | 22889528 | 22913629 | 0.02 | 3 (0.007) | Multigenic |
| 22q11.22 | Deletion | 23059563 | 23224058 | 0.16 | 11 (0.02) | Non-genic region |
| 22q11.23-q12.1* | Duplication | 25661725 | 25910667 | 0.24 | 25 (0.06) | IGLL3P, LRP5L, CRYBB2P1 |

*Found in SWI
